# Comprehensive genomic analysis of antibiotic resistance plasmids in animal-associated *Staphylococcus aureus* in France

**DOI:** 10.1128/spectrum.00772-25

**Published:** 2025-09-18

**Authors:** Rachel Contarin, Séverine Murri, Antoine Drapeau, Tom Cayssials, Jean-Yves Madec, Emilie Dordet-Frisoni, Marisa Haenni

**Affiliations:** 1 INTHERES, Université de Toulouse, INRAE, ENVT 27091, Toulouse, France; 2 Anses–Université de Lyon, Unité Antibiorésistance et Virulence Bactériennes, Lyon, France; Universidade de Sao Paulo, Sao Paulo, Brazil

**Keywords:** *Staphylococcus aureus*, plasmids, antibiotic resistance, MGE, HGT, veterinary

## Abstract

In *Staphylococcus aureus*, an animal pathogen and zoonotic agent, plasmids play a pivotal role in the acquisition and spread of antibiotic resistance genes (ARGs). This study investigated the plasmid content of 329 *S*. *aureus* isolates from livestock and companion animals collected in France between 2010 and 2021. Plasmids (*n* = 211) were identified from 139 isolates. The major families identified—*rep7a*, *rep20*, and *rep10*—were associated with specific resistance genes (*str*, *cat*, *blaZ*, *erm*(C)) and exhibited widespread horizontal transfer across different *S. aureus* sequence types (STs) and animal hosts. In temporal analysis, the *rep7a*/str and *rep7a*/*cat* plasmids circulating in horses were progressively replaced by a *rep7a* plasmid carrying both *str* and *cat* genes. The study also highlighted the presence of mosaic plasmids, which combined elements from different bacterial species/genera, confirming the broad host range of *S. aureus* plasmids and their ability to acquire ARGs from diverse sources. Moreover, the occurrence of hybrid plasmids (carrying multiple *rep* genes) underscores the plasticity of these vectors of ARGs. This study emphasizes the need to investigate the mechanisms driving the spread and persistence of antibiotic-resistant plasmids in *S. aureus*, with a view to developing strategies aimed at combating antibiotic resistance.

**IMPORTANCE:**

The spread of antibiotic resistance in *Staphylococcus aureus* is a growing concern, particularly in animals that can serve as reservoirs for resistant strains. This study highlights the crucial role of plasmids in transmitting resistance genes among different animal hosts and *S. aureus* lineages. The characterization of 329 isolates collected over 10 years revealed how certain plasmid families are associated with specific resistance genes and how they evolve over time. The occurrence of mosaic and hybrid plasmids further underscores the ability of *S. aureus* to acquire resistance from diverse bacterial sources. These findings provide key insights into the mechanisms shaping antibiotic resistance in this pathogen and emphasize the fact that understanding plasmid-driven resistance is essential for developing effective interventions to limit the spread of multidrug-resistant *S. aureus* in both veterinary and human medicine.

## INTRODUCTION

*Staphylococcus aureus* is an opportunistic pathogen that triggers infections and diseases in numerous animal hosts (1) and poses a significant zoonotic threat. As a result, it is classified among high-priority bacteria by the World Health Organization (WHO) and the World Organization for Animal Health (WOAH). Under selective pressure, *S. aureus* can acquire numerous antibiotic resistance genes (ARGs) (2), the best known being the *mecA* gene, which confers resistance to all β-lactam antibiotics. Resistance gene dissemination is often mediated by mobile genetic elements (MGEs), which account for 15–20% of the *S. aureus* genome (3). In a recent study, plasmids, as key components of the *S. aureus* mobilome, were identified as the primary carriers of ARGs (4). These MGEs are small extrachromosomal circular DNA molecules that can replicate independently of the host chromosome and efficiently transfer resistance and virulence determinants among bacteria, even across species (3). Although extensively studied in Enterobacterales, they have long been underestimated in Gram-positive bacteria, including staphylococci.

Until recently, conjugation was considered a relatively rare event in *S. aureus*, as only about 5% of staphylococcal plasmids encode the machinery required for autonomous transfer (5); however, conjugative plasmids can facilitate the transfer of mobilizable plasmids present in the same cell (6, 7). Mobilizable plasmids either carry an *oriT* and encode a corresponding relaxase or only carry an *oriT* that is recognized by the relaxase of a conjugative plasmid. In order to be transferred, these relaxases must recognize the coupling protein of a conjugative plasmid, which actively promotes their transfer (2, 8, 9). Small plasmids < 45 kb, which are by far the most numerous in *S. aureus*, can also be transferred by generalized transduction, while natural genetic competence, although of lesser importance, might also play a role in plasmid transfer (10, 11).

Staphylococcal plasmids are classified into three classes according to their size and replication mechanism: (i) small (1.3–4.6 kb) multicopy plasmids, cryptic or carrying a single resistance determinant; (ii) larger (15–30 kb) low copy plasmids, which usually carry several resistance determinants; and (iii) conjugative (30–60 kb) multi-resistance plasmids (12–14). Larger plasmids undergo theta replication, whereas small plasmids usually replicate via an asymmetric rolling-circle mechanism (15, 16). Staphylococcal plasmids are also classified based on the nucleotide sequence of conserved regions of replication-initiating (*rep*) genes. Recurrent associations between *rep* genes and ARGs are described, such as *rep7* associated with tetracycline resistance, *rep10* associated with macrolide resistance, and *rep16* associated with β-lactam resistance (17). A PCR-based replicon typing (PBRT) scheme has been developed to classify plasmids into enterococci and staphylococci, encompassing 26 *rep* families and 10 unique families (18, 19). This classification is a useful tool for investigating plasmid dynamics within bacterial populations across different ecological niches (20).

The present study, in line with recent findings on the role of plasmids as key players in the spread of ARGs, aimed to characterize the diversity of ARG-carrying plasmids circulating among pathogenic *S. aureus* isolates from livestock and companion animals in France. Plasmids from field *S. aureus* isolates collected through the Resapath network (21) were fully sequenced, and genomic data were analyzed alongside associated metadata, including host, date of sampling, and geographical origin. A subset of 81 isolates displaying multiple ARGs was further whole genome-sequenced to localize precisely all of the identified ARGs. This approach enabled the identification of predominant and sporadic plasmid-ARG associations and provided insights into their dissemination dynamics.

## MATERIALS AND METHODS

### Bacterial isolates

This study included 329 *S*. *aureus* isolates of veterinary origin collected between 2010 and 2021 via the French Resapath network (21) across 70 different French departments. Isolates were collected from horses (*n* = 90), dogs (*n* = 87), cats (*n* = 83), cattle (*n* = 44), sheep (*n* = 11), goats (*n* = 3), primates (*n* = 4), poultry and birds (*n* = 3), and rodents (*n* = 4) (Table S1). The isolates included in this study were selected from a larger collection (*n* > 2,000 isolates). Among these >2,000 isolates, 525 were collected between 2010 and 2021. From these 525 isolates, our final collection of 329 isolates was chosen to be representative of the French territory, different years, and different resistance profiles found in this collection. All isolates were cultured on a COS medium (Biomérieux, Marcy l’Etoile, France) at 37°C.

### Antibiotic susceptibility testing

Susceptibility testing was performed using the disk diffusion method on Mueller-Hinton agar in accordance with the guidelines of the Antibiogram Committee of the French Society for Microbiology (22). *S. aureus* ATCC 25923 was used as the quality control strain. The antibiotics tested included penicillin G (PEN), kanamycin (KAN), gentamicin (GEN), tobramycin (TOB), chloramphenicol (CHL), florfenicol (FFL), tetracycline (TET), erythromycin (ERY), fusidic acid (FD), cefoxitin (FOX), spiramycin (SPI), lincomycin (LIN), and enrofloxacin (ENR) (Mast Diagnostics, Amiens, France).

### Detection of antimicrobial resistance genes

The 30 most common ARGs in *S. aureus* of animal origin were identified using the multiplex PCR technique (Table S2) (4). Genes not detected by PCR but identified through sequencing were included in the analysis. DNA extractions were performed using the boiling method, heating colonies at 100°C for one hour in one mL of NaCl 0.9%. PCRs were performed using the Qiagen Multiplex PCR Kit (Qiagen, Courtaboeuf, France) in a final volume of 25 µL. The following program was used for all multiplex PCRs: initial lysis and denaturation step at 95°C for 15 min, followed by 35 cycles at 94°C for 30 s, 55°C for 30 s (annealing), and 72°C for one minute. A final extension step was performed at 72°C for ten minutes. The amplicon sizes ranged from 158 to 1272 bp.

### Plasmid detection

The presence of plasmids was assessed using pulsed-field gel electrophoresis (PFGE) on S1-digested DNA (Fig. S1), as detailed in the Supplementary material.

For isolates in which no plasmid was detected by PFGE (*n* = 114), the presence of plasmids was verified by dot blot hybridization using a probe panel targeting conserved fragments of the *rep* genes labeled with digoxigenin by PCR using the primers listed in Table S3. Drops (4 µL) of boiled genomic DNA from each isolate were spotted in duplicate onto positively charged nylon membranes (Hybond N1; Amersham Biosciences, Orsay, France). Rep probes were hybridized and detected using a DIG-HIGH Prime DNA Labeling and Detection Starter Kit II (Roche Diagnostics, Meylan, France) following the manufacturer’s instructions.

### Short- and long-read sequencing

A subset of 81 isolates chosen among the isolates carrying the largest number of ARGs and with diverse PFGE and resistance profiles (identified by PCR) was short-read whole genome-sequenced. DNA was extracted using the NucleoSpin Microbial DNA Extraction Kit (Macherey-Nagel, Hoerdt, France). For all other isolates presenting at least one plasmid via S1-PFGE (*n* = 142) or dot blot (*n* = 4), plasmid DNA was extracted using a Plasmid DNA Purification NucleoBond PC20 Kit (Macherey-Nagel) and long read-sequenced. Each identified plasmid was designated with a 'p,' followed by the corresponding strain number. In cases where multiple plasmids were detected within the same strain, they were numbered in a decreasing order of size, with the largest plasmid assigned to number 1 and the smallest assigned to the highest number. Library preparation, short-read whole-genome sequencing (Illumina NovaSeq6000 technology), and long-read plasmid sequencing (GridION Oxford Nanopore Technology, Oxford Nanopore Technology, United Kingdom) were outsourced to Eurofins Genomics (Ebersberg, Germany). Short reads were quality trimmed and *de novo* assembled using Shovill v1.0.4, and the quality of assemblies was assessed using QUAST v5.0.2 (23) (Table S4). With regard to plasmids, *de novo* assembly was performed using either Unicycler v0.5.0 (24) or Flye v2.9.2 (25). Plasmids were annotated using Bakta 1.9.1 software (DB: 5.0.0).

### Characterization of plasmids and genomes and phylogenetic analysis

Plasmid replicons (*rep* coding gene) were detected using PlasmidFinder v.2.1.6 software (databases: v.2021-11-29) with default parameters (26, 27). Conjugation systems and proteins involved in mobilization were identified using CONJscan (28, 29), MOBscan (30), and Bakta 1.9.1 software (DB: 5.0.0). The presence of *oriT* was detected using the latter (DB: 5.0.0). ARGs were identified using ResFinder v.4.1.7 (database: v.2021-09-22) with default parameters (26, 31) and RGI-Comprehensive Antibiotic Resistance Database (CARD) (32). MinHash Approximation of SHared k-mers (MASH) v2.2.2 was used to calculate the distance between nucleotide sequences of plasmids using default parameters (33). Plasmid clustering was performed with thresholds of 0.18 for MASH clusters (A to N) and 0.06 for MASH groups defined by a numeric identifier (34). Results were visualized using iTOL v7 (https://itol.embl.de).

Genomes and MGEs, including integrative and conjugative elements (ICEs), transposons, composite transposons, SCC*mec*, and phages, were characterized as previously described (4). The *spa* type was determined using SpaTyper v0.3.3 (35). A neighbor-joining tree based on the *S. aureus* core genome (cgMLST), with core gene data available at https://www.cgmlst.org/ncs (36), was constructed using pyMLST v.2.1.3 with default parameters (37) and visualized using iTOL v7 (https://itol.embl.de).

## RESULTS

### Diversity of resistance genes in *S. aureus* isolated from animals

Analysis of the resistome of the 329 *S*. *aureus* veterinary clinical isolates collected between 2010 and 2021 via the Resapath network (21) showed that 92% (*n* = 303) carried at least one of the tested ARGs, with a maximum of 11 ARGs in a single isolate and an average of 4.3 genes per isolate. Among the 30 genes tested, 26 were detected (Table S2). The high prevalence of *mecA* (269/329; 82%) and *blaZ* (231/329; 70%), both conferring resistance to β-lactams, reflected a sampling bias due to the laboratory collection, which mostly counted MRSA isolates (Table S1; Fig. S2). In addition to these two genes, the *tet*(M) (tetracycline) (179/329; 54%), *aac(6′)-aph(2*′) (aminoglycosides) (131/329; 40%), and *dfrK* (trimethoprim) (111/329; 34%) resistance genes were the most frequently identified (Table S1; Fig. S2). Overall, no significant host specificity was observed (Fig. S2), except for the *aac(6′)-aph(2*′) gene, which was overrepresented in isolates from equines (91%) and primates (75%), and the *dfrK* gene, which was significantly more abundant in equine isolates (77%) (Fig. S2). The observed genotypes were consistent with the resistance phenotypes determined by disk diffusion (Table S1; Fig. S3).

### Plasmid diversity in *S. aureus* isolates of animal origin

PFGE on S1-digested DNA was performed on 256 isolates carrying at least one ARG without taking into account the chromosomally encoded *mecA* gene. Among them, 139 isolates (139/256; 54%) harbored at least one plasmid as determined by PFGE (Table S1). Long-read sequencing (Oxford Nanopore) of these 139 isolates revealed the presence of 211 plasmids carrying replicon proteins, since 54 isolates (39%) contained multiple plasmids (up to five, including six isolates presenting two plasmids of the *rep7a* family) (Table S1). Twenty-one hybrid plasmids also referred to as “multi-replicon plasmids” were identified, which carried two (*n* = 19) or three (*n* = 2) *rep* genes (Table S5). The 211 plasmids ranged in size from 1.4 to 50.6 kb, with 156 (74%) varying from 1.4 to 8.9 kb, and 55 (26%) from 19.7 to 50.6 kb (Fig. S4). Twenty-five isolates harboring multiple plasmids carried both large (>20 kb) and small (<10 kb) plasmids.

**Fig 1:**
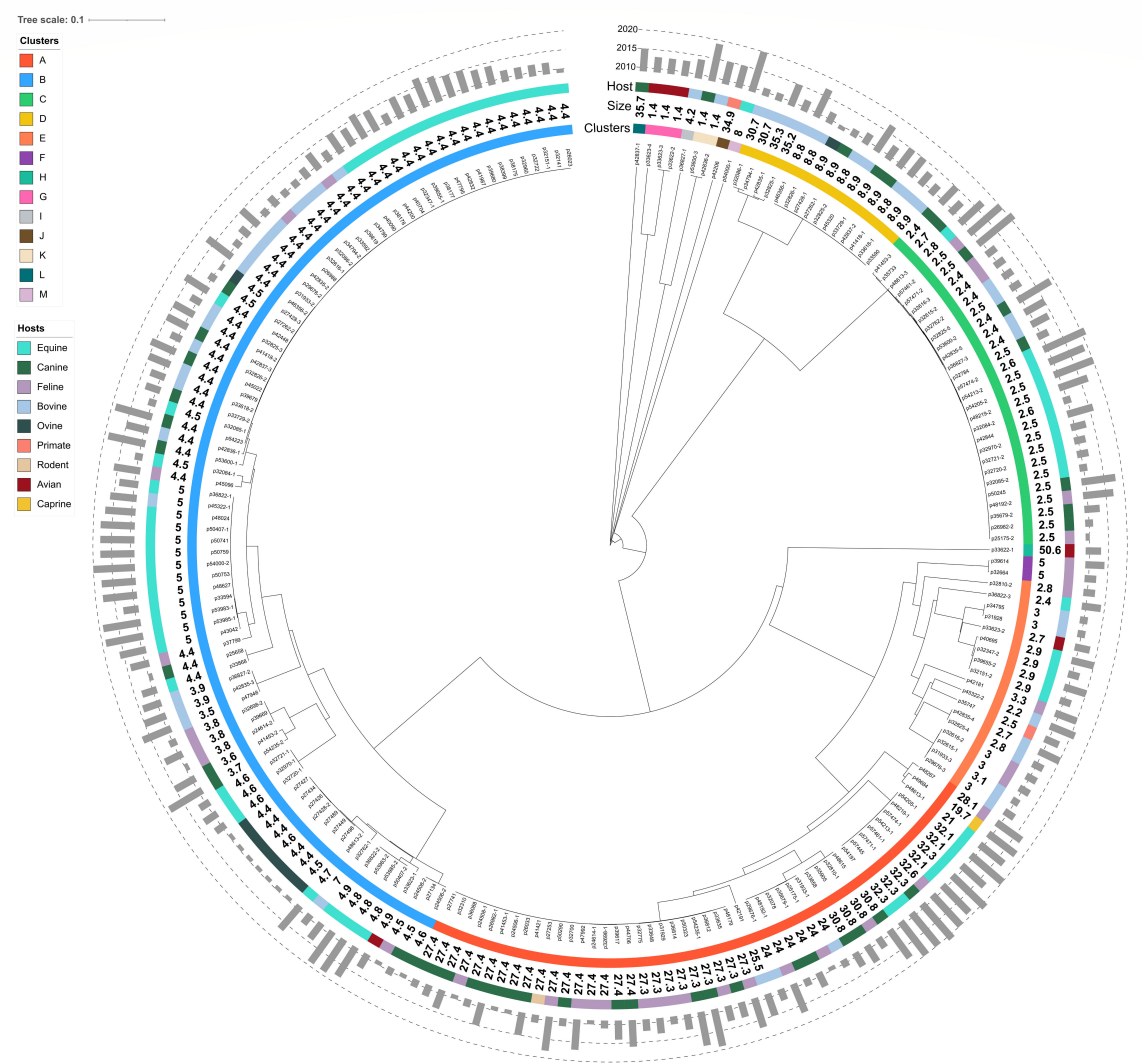
MASH analysis of the 211 plasmids from *S. aureus* isolates. This tree based on MASH distances was constructed using the neighbor-joining method and visualized using iTOL v7 (https://itol.embl.de). From the outer to the inner circle, the isolation year and the host of the isolate carrying a plasmid, size of the plasmid, and clusters are defined with a threshold of 0.18 MASH distance. The correspondence between colors and each host or cluster type is indicated in the top left corner.

Resistome analysis of all plasmids revealed that 91% (*n* = 192/211) encoded at least one ARG (Table S5). MASH-based clustering grouped all plasmids into 13 heterogeneous clusters (named A to M) (Fig. 1), with clusters A to C being predominant (see sections below for detailed description). Most plasmids from clusters E to N either lacked ARGs or represented rare and atypical plasmids (Table S5).

#### Plasmids of the *rep20* family

**Fig 2:**
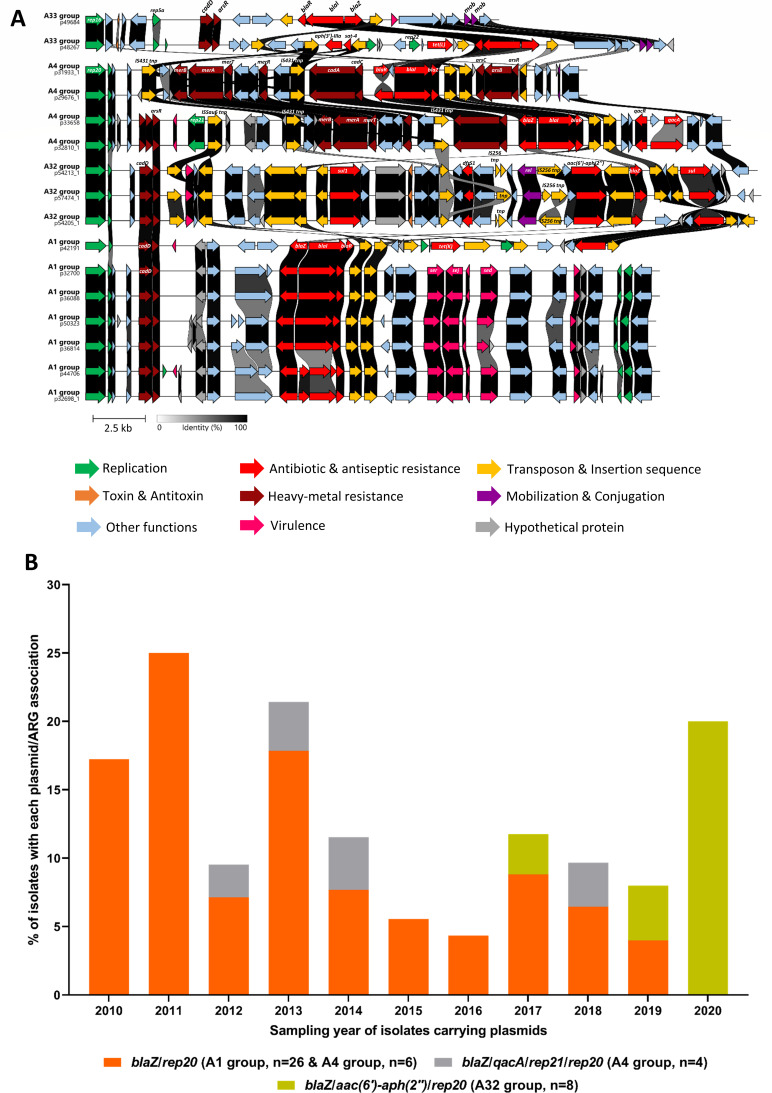
Plasmids of the *rep20* family. (**A**) DNA sequence comparison of plasmids belonging to the MASH cluster A (using the Clinker web-based tool https://cagecat.bioinformatics.nl/tools/clinker). The group to which each plasmid belongs and its name are indicated in front of each sequence. Only selected representatives from each group of MASH cluster A are shown. The nucleotide sequence identity between each coding region is represented with a color gradient ranging from white (0% homology) to black (100% homology). The scales for the gradient and size are located at the bottom. Genes of interest are highlighted in color to specify their function, which is indicated in the legend provided at the bottom. A few genes of interest are also specified. rel: relaxase; mob: mobilization protein. (**B**) Histogram showing the frequency of isolates carrying each plasmid from the *rep20* family, along with their associated ARGs, according to the year the plasmid-carrying strain was isolated. Each plasmid/ARG association is represented by a different color, as detailed in the legend at the bottom. In addition to the ARGs and *rep* families carried by the plasmids, the group and the number of isolates carrying each plasmid are indicated alongside each color.

Cluster A comprised 48 plasmids (23%), most of which harbored the *rep20* gene (Table S5). Among these, four plasmids carried the *rep20* gene in association with *rep21*. Additionally, four plasmids carried multiple *rep* genes: one with a *rep16*/*rep19*/*rep7a* combination, another with *rep16*/*rep22*/*rep5a*, and two with *rep16*/*rep5a* (Table S5). Based on genomic distances calculated by k-mer analysis using MinHash Approximation of SHared k-mers (MASH) (33), cluster A was further divided into four groups (A1, A32, A4, and A33) according to the plasmid size, associated ARGs, and accessory genes present on the plasmid backbone (Fig. 2A).

The A1 group encompassed 27 large plasmids (25–27 kb), all of which carried *rep20*, *oriT,* and *blaZ* genes, except for the p42191 plasmid, which additionally carried the *tet*(K) gene, a relaxase MOBV, and belonged to *rep16*/*rep19*/*rep7a* families (Table S5). This *tet*(K) gene was located on a composite transposon formed by two IS*Sau6* elements (Table S5). All plasmids in this A1 group carried the *cadD* gene (low-level cadmium resistance). Apart from p42191, they also carried the enterotoxin genes *sed*, *ser*, and *sej* (Fig. 2A), which are known to be plasmid-borne and are frequently reported in clinical isolates (38, 39). A1 group plasmids were identified between 2010 and 2019 (Fig. 2B) in both companion (cats, dogs, and a rabbit) and livestock (cattle) animals (Table S5; Fig. 1).

The A32 group comprised eight large (32 kb) plasmids carrying the *blaZ*, *sul1*, *dfrS1*, and *aac(6′)-aph(2*″) resistance genes (Table S5; Fig. 2A), as well as an *oriT* and a gene encoding a relaxase (Fig. 2A). This relaxase gene was flanked by two IS*256* insertion sequences, suggesting the formation of a composite transposon and the potential mobility of this region. These plasmids were almost exclusively detected in the *S. aureus* isolates of equine origin (*n* = 6) and observed in 2017, 2019, and 2020. In 2020, A32 group plasmids were the only ones detected from cluster A, suggesting a recent and potentially expanding emergence of this plasmid lineage in horse-associated isolates across France (Fig. 2B; Table S5).

The A4 group (*n* = 10) comprised plasmids carrying the *mer* operon (*merR*, *merA*, *merB*, *merT*), which was consistently flanked by two IS*431* insertion sequences, suggesting the potential mobility of this heavy metal resistance region (Fig. 2A). Based on plasmid content, this group could be further split into two subgroups. The first (*n* = 6) comprised 24 kb plasmids (e.g., p31933_1 and p29676_1 in Fig. 2A) carrying conjugal transfer protein, the *blaZ* gene, and harbored by strains from cattle, cats, and dogs from 2010 to 2017 (Table S5). The second group (*n* = 4) included larger *rep20*/*rep21* hybrid plasmids (31 kb) (e.g., p33658 and p32810_1 in Fig. 2A) displaying an *oriT*, the *blaZ* gene, and the *qacA* gene conferring resistance to biocides (Table S5). These plasmids were exclusively detected in companion animals between 2012 and 2018 and showed no evident temporal or spatial link with other *rep20* plasmids (Table S5; Fig. 2B).

The last group of cluster A, A33, included three hybrid plasmids, namely, p48613-1 (20 kb), p49684 (21 kb), and p48267 (28 kb), which all displayed an *oriT* and proteins involved in mobilization (Fig. 2A; Table S5). The two first plasmids carried the *rep16*/*rep5a* and the *blaZ* genes, while p48267 additionally harbored a genetic region carrying three other antibiotic resistance genes (*aph(3')-III*, *tet*(L), and *sat-4*), along with a replication gene from the *rep22* family (Fig. 2A).

Globally, the proportion of plasmids carrying only the *blaZ* gene (groups A1 and A4) tended to decline from 25% in 2011 to less than 5% in 2019 and was no longer detected in 2020 in this data set (Fig. 2B). In parallel, since 2017, a new *rep20* plasmid (A32 group) carrying both *blaZ* and *aac(6′)-aph(2*″) genes has emerged across France, predominantly in *S. aureus* isolated from horses (Fig. 2B; Table S5).

#### Plasmids of the *rep7a* family

**Fig 3:**
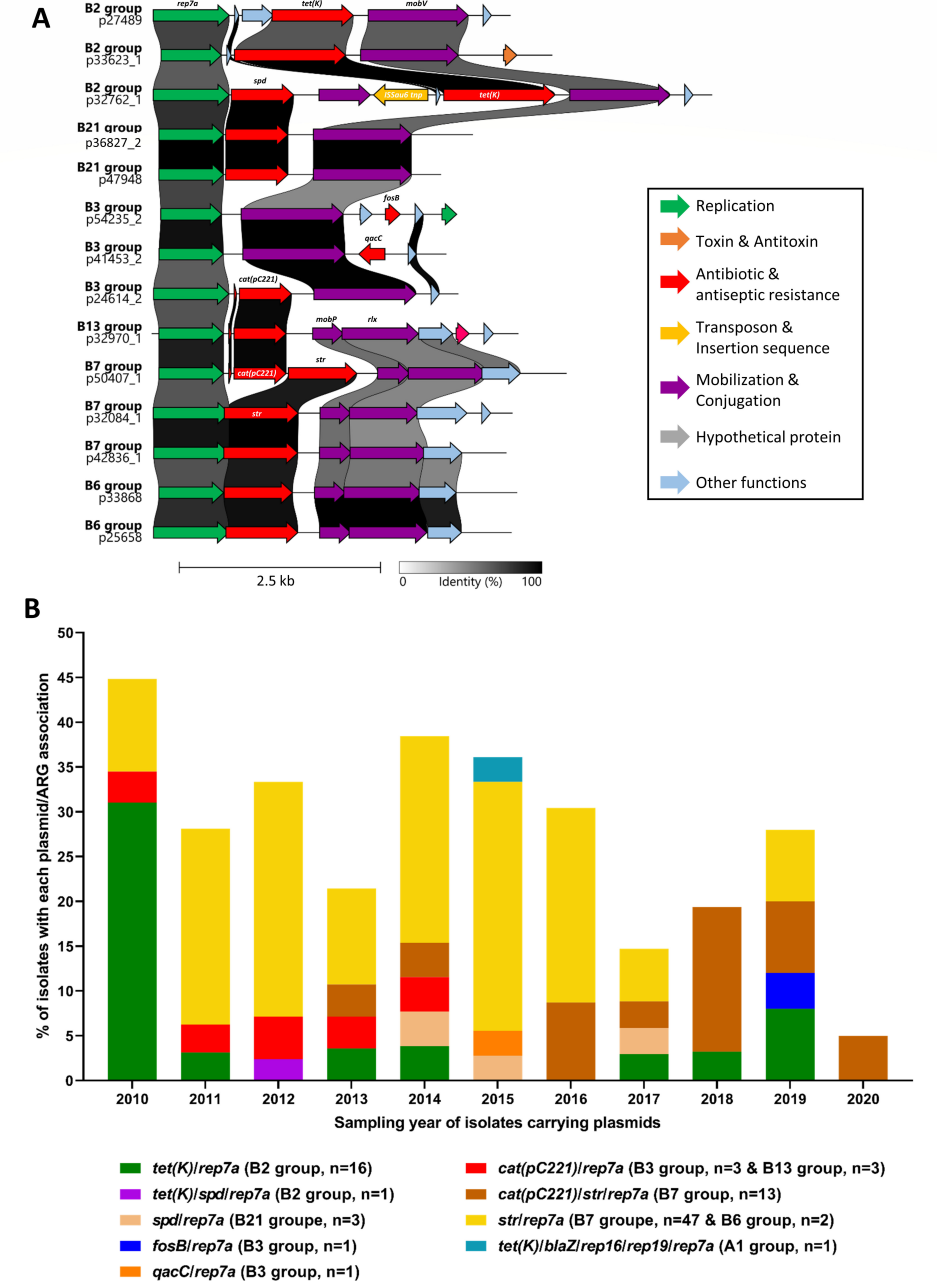
*rep7a* plasmid family. (**A**) DNA sequence comparison of plasmids belonging to the MASH cluster B (using the Clinker web-based tool https://cagecat.bioinformatics.nl/tools/clinker). The group to which each plasmid belongs and its name are indicated in front of each sequence. Only selected representatives from each group of MASH cluster B are shown. The nucleotide sequence identity between each coding region is represented with a color gradient ranging from white (0% homology) to black (100% homology). The scales for the gradient and size are located at the bottom. Genes of interest are highlighted in color to specify their function, which is indicated in the legend provided at the bottom. A few genes of interest are also specified. mob: mobilization protein. (**B**) Histogram showing the frequency of isolates carrying each plasmid from the *rep7a* family, along with their associated ARGs according to the year the plasmid-carrying strain was isolated. Each plasmid/ARG association is represented by a different color, as detailed in the legend at the bottom. In addition to the ARGs and *rep* families carried by the plasmids, the group and the number of isolates carrying each plasmid are indicated alongside each color.

Cluster B encompassed 90 plasmids (43%), making it the most prevalent cluster identified in this study. All of these plasmids carried only the *rep7a* gene and were small in size, ranging from 3.5 to 5 kb, with one outlier of 7 kb. This cluster was divided into six groups based on MASH analysis (B7, B2, B3, B6, B13, and B21), taking into account both plasmid backbone structures and the ARGs they carried (Fig. 3; Table S5).

The B7 group was the largest (*n* = 60) and comprised plasmids sharing a high sequence similarity (Fig. 1 and 3A). These plasmids either carried only the *str* gene (*n* = 47) (e.g., p32084-1 and p42836-1) or both the *str* and *cat(pC221*) genes (*n* = 13) (e.g., p50407-1), conferring resistance to streptomycin and chloramphenicol, respectively (Fig. 3A; Table S5). All plasmids also encoded proteins involved in mobilization (MOBP), and most of them harbored an *oriT* (40/60) (Table S5; Fig. 3A). They were identified in different animal hosts, including cats, dogs, horses, cattle, and goats, over the entire study period from 2010 to 2020 (Table S5; Fig. 3B).

The B2 group encompassed 17 plasmids mostly carrying the *tet*(K) gene. Apart from p53983-2, all of them also encoded the mobilization protein MOBV (Table S5). One plasmid, p32762-1 (7 kb), also harbored the *spd* gene (*ant (9*) gene family) conferring resistance to spectinomycin, along with the insertion sequence IS*Sau6* (Table S5; Fig. 3A). The B3, B6, B13, and B21 groups comprised two to five plasmids, which carried various ARGs, including *cat(pC221)* (e.g., p24614-2 from B3 and p32970-1 from B13), *str* (e.g., p33868 and p25658 from B6), *fosB* (e.g., p54235-2 from B3), *spd* (e.g., p36827-2 and p47948 from B21), and *qacC* (biocide resistance) (e.g., p41453-2 from B3) (Fig. 3A; Table S5).

Temporal analysis of this sampling of *rep7a* plasmids showed that, until 2012, the *str* and *cat(pC221*) genes were carried on distinct plasmids, primarily in equine isolates (Fig. 3B), with the *rep7a*/*cat(pC221*) plasmid mostly found in the Parisian region (Table S5). From 2013 onwards, a plasmid carrying both genes emerged in the western Paris area and progressively replaced the plasmids carrying individual resistance genes (Fig. 3B; Table S5).

#### Plasmids associated with *erm*(C) genes: *rep10* family and others

Cluster C encompassed 28 plasmids (13%) of very small size (2.4–2.8 kb), all of which belonged to the *rep10* family. These plasmids exhibited highly conserved sequences with no mobilization protein and no *oriT* and consistently carried only the *erm*(C) gene, conferring resistance to macrolides (Table S5). They were identified in companion animals, horses, and cattle from 2010 to 2020.

Apart from *rep10* plasmids, the *erm*(C) gene was also detected on hybrid plasmids belonging to cluster D (*n* = 15). Plasmids from the D8 group as defined by the MASH analysis (*n* = 11, 8.7–8.9 kb) harbored a *repUS18*/*repUS12* replication module and carried multiple resistance determinants, including the *erm*(C), *aadD*, and *tet*(L) genes (Table S5). Notably, a majority of these plasmids from the D8 group harbored the protein mobilization MOBV (*n* = 7/11); they were isolated from cattle, dogs, and goats between 2010 and 2017 (Table S5).

#### Atypical plasmids

The 30 plasmids belonging to clusters E to M (14.2%) exhibited significant sequence variability. Cluster E exclusively comprised plasmids harboring *rep13* or *rep21* genes, ranging in size from 2.2 to 3.3 kb (Table S5), and encoding two to six coding sequences. Only a few of these plasmids carried resistance genes: *cat*(pC194) was found in four *rep13* plasmids (e.g., p32151-2, p40695), *lnu(A*) in one (p33623-2), and *qacC*, *qacJ*, or *qacG* in four *rep21* plasmids (e.g., p35747, p32825-4, p42835-4, and p45322-2) (Table S5). The functions of the remaining coding sequences were mostly unknown.

Two large atypical ARG-carrying plasmids each constituted a unique cluster: p42837-1 (cluster L) and p33622-1 (cluster H) (Table S5; Fig. S3). The p42837-1 plasmid resembled those carrying the *cfr* gene (97.69% identity (85% query cover) with the pSA737 plasmid [40] and 97.12% (query cover 92%) with the p12-02300 plasmid of *Staphylococcus epidermidis* [41]) and thus likely belonged to the pWBG4-type conjugative plasmid family (5). This plasmid harbored the Tn*558* transposon carrying the *fexA* gene but lacked the typical 3 kb region flanking the *cfr* gene, with truncated *tnrB* and *bin3* genes (Fig. S5A). The p33622-1 plasmid appeared to be a hybrid between *S. aureus* and *Enterococcus faecium* plasmids (Fig. S5B). The successful circularization of the plasmid confirmed its mosaic structure and circular form, but the PlasmidFinder software used in this study was unable to detect any replication gene families. The *S. aureus* portion spanning over 30 kb with a 31% GC content was very similar to plasmid pM084526_1 (99.7% identity, 83% coverage for this region), originating from a human ST398 isolate (RIVM_M084526) (42) and carrying the *fexA* and *cfr* genes. However, in p33622-1, only the region containing the putative T4SS system was conserved, with no resistance gene retained (Fig. S5B). Additionally, in the *S. aureus* region, the plasmid included a PemIKSa toxin-antitoxin system previously described in plasmid pCH91 (43). In p33622-1, 38% of the sequence appeared to derive from *E. faecium* (CP091208.1) or *E. durans* (CP043327.1) plasmids, with >99.9% nucleotide identity. This region harbored the *erm(B*) gene, a zeta toxin-antitoxin system, and the IS*1216* insertion sequence commonly associated with the *cfr* gene in *Enterococcus* plasmids (44).

Additional atypical plasmids were detected as well, including p42206 (*rep24b*), p54000-1 (*rep5c*), p36827-1 (ND, undetermined family), and p32664 (*rep5d*), none of which were associated with ARGs (Table S5). Notably, a conjugation system similar to that of plasmid pGO1 (45) (T4SS_typeFATA) was detected in plasmid p44206 (Table S5). These plasmids generally originated from human-associated *S. aureus* or, in the case of the latter two, from coagulase-negative staphylococci, which are known reservoirs of MGEs and potential sources of ARGs.

### Plasmid dissemination between sequence types

**Fig 4:**
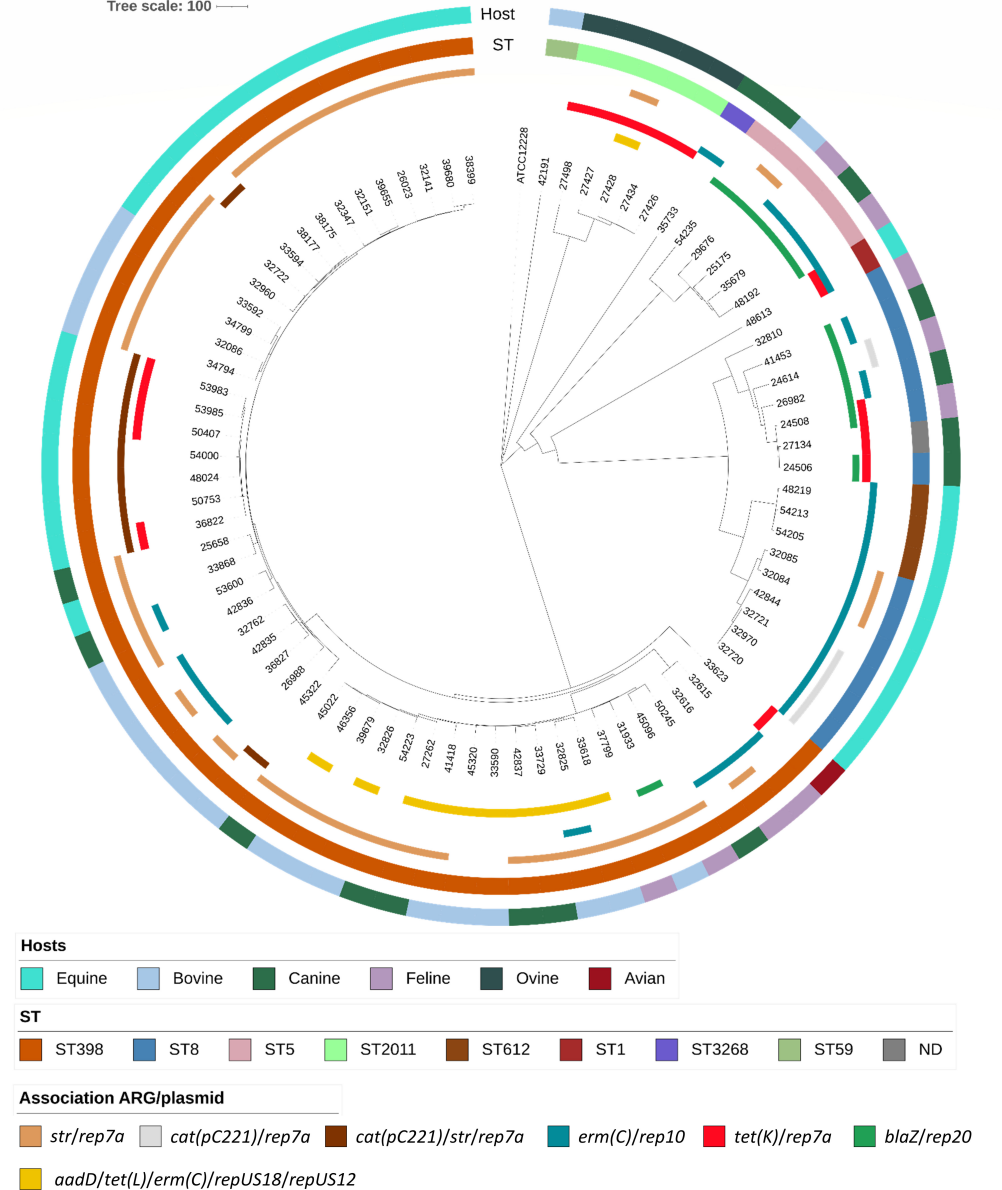
Phylogenetic tree of 81 *S*. *aureus* isolates sequenced using Illumina technology and occurrence of the main plasmids carrying antibiotic resistance genes (ARGs). This tree based on the cgMLST distance was constructed using the neighbor-joining method and visualized using iTOL v7 (https://itol.embl.de). From the outer to the inner circle, the annotations represent the host, sequence type (ST), and diverse ARG/plasmid associations. The correspondence between colors and each host type, ST, or ARG/plasmid association is indicated on the right. The tree was rooted using the *Staphylococcus epidermidis* strain ATCC12228.

A subset of 81 isolates that presented the highest number of plasmids carrying ARGs was whole genome-sequenced. A large proportion of the isolates (52/81; 64%) belonged to ST398, followed by ST8 (12/81; 15%) and ST5 (5/81; 6%) (Table S1; Fig. 4). The ST398 strains comprised isolates of eight different *spa* types, including *t011*, which is typically associated with horses, and carried two SCC*mec* types (IV and V) (Table S1). Within each ST, several of the ARG/plasmid associations described above (e.g., *str*/*rep7a*, *tet*(K)/*rep7a*, *erm*(C)/*rep10*, or *aadD*/*tet*(L)/*erm*(C)/*repUS18*/*repUS12*) were identified (Fig. 4; Table S5). For instance, ST8 isolates displayed 24 plasmids, and ST398 isolates carried 94, including primarily *erm*(C)/*rep10* (*n* = 8) and *str*/*rep7a* (*n* = 36), respectively (Table S5; Fig. 4). In addition to being clonally expanded within each ST, several ARG/plasmid associations were identified across multiple STs, including *erm*(C)/*rep10* in six STs, *str*/*rep7a* and *tet*(K)/*rep7a* in four, *blaZ*/*rep20* in three, and *aadD*/*erm*(C)/*tet*(L)/*repUS18*/*repUS12* in two (Table S5; Fig. 4).

### Plasmid versus chromosomal location of antibiotic resistance genes

Whole-genome sequencing revealed distinct localization patterns for ARGs. Several genes, namely, *spd*, *str*, *lnu(A*), *erm(C*), *erm(T*), *cat(pC221*), *cat(pC194*), and *qac*, were exclusively plasmid-borne (Table 1; Fig. S6A), while others like *aac(6′)-aph(2*″), *ant (6)-Ia*, *ant (9)-Ia*, *aph(3′)-III*, *fusA*, *fusC*, *SA fosB*, *lnu(B*), *lsa(E*), *vga(A)V*, *dfrC*, and *dfrK*, were predominantly chromosomal (Table 1). The *aadD* gene was identified either on the chromosome or on plasmids: it was mostly plasmid-borne in whole-sequenced genomes (15/26; 58%) (Table 1) but was less (15/59, 25%) associated with plasmids among the 329 analyzed isolates (Fig. S6B). When plasmid-associated, it co-occurred with *erm(C*) and *tet(L*). The *blaZ* gene was also largely chromosomal (58/75; 77%), with the plasmid variant (mostly on *rep20* plasmids) declining over time. Notably, chromosomal and plasmid *blaZ* variants differed in sequence (Table 1; Fig. S6C).

**TABLE 1:** Association of antibiotic resistance genes (ARGs) with plasmids or chromosome and with other mobile genetic elements (MGEs)^*a*^

Antibiotic family	ARGs	Chromosome	Plasmid	Other MGEs
Aminoglycoside	*aac(6')-aph(2''*)	33	1	
			2	cn_IS*256*
	*aadD*	5	11	
			4	cn_IS*Sau6*
		6		cn_IS*Sau6*
	*ant (6)-Ia*	4		
	*ant (9)-Ia*	1		
		3		Tn*554*
	*aph(3')-III*	2		
	*sat-4*	1		
	*spd*		3	
	*str*		49	
Fosfomycin	*fusA*	2		
	*fusC*	1		
	*SA fosB*	22		
Lincosamide	*lnu(A*)		1	
	*lnu(B*)	2		
	*lsa(E*)	2		
	*vga(A)V*	4		Tn*5406*
Macrolide	*erm(A*)	1		
		3		Tn*554*
	*erm(B*)	1		
		1		Tn*917*
	*erm(C*)		35	
	*erm(T*)		2	cn_IS*Sau6*
			2	
Phenicol	*cat(pC194*)		3	
	*cat(pC221*)		13	
	*cat(pC233*)	1		
	*fexA*	13	1	Tn*558*
Tetracycline	*tet(K*)	8	15	
			1	cn_IS*Sau6*
	*tet(L*)	1	11	
			4	cn_IS*Sau6*
	*tet(M*)	60		Tn*916*
Trimethoprim	*dfrC*	11		
		15		cn_IS*Sau6*
	*dfrK*		2	
		27		Tn*559*
β-Lactam	*blaZ*	58	17	
		1		Tn*552*
	*mecA*	69		
		7		cn_IS*Sau6*
Antiseptic	*qacA*		1	
	*qacC*		1	
	*qacG*		1	
	*qacJ*		2	

^*a*^ For each ARG, the number of genes associated with either the chromosome or plasmid is indicated. Other MGEs include composite transposons (elements beginning with “cn”), transposons (TnX), or an ICE (Tn916).

Some chromosomal ARGs were embedded in MGEs facilitating their spread. For example, *fexA* and *dfrK* were consistently linked to transposons *Tn558* and *Tn559*, respectively, regardless of the genomic location. ICEs and prophages were also abundant, but none carried ARGs: 83 ICEs (Tn916/*tet(M*) and ICE6013) were identified in 64 genomes (79%) (Table 1; Table S1), and 155 prophages from 18 families were found in 77 genomes (95%).

## DISCUSSION

The role of plasmids in the dissemination of ARGs among Gram-positive bacteria remains less explored than that in Gram-negative bacteria. A recent study highlighted plasmids as the main vectors of ARGs in *S. aureus* (4). Since this study relied entirely on National Center for Biotechnology Information (NCBI) data sets, conclusions about the prevalence and evolution of these elements might suffer from the inherent limitations of this type of large public database (i.e., strong sampling biases, incomplete or imprecise metadata, and the frequent embedding of plasmid sequences within draft genome assemblies), thus making accurate plasmid identification and characterization more complicated. To better describe the diversity and dynamics of circulating plasmids, our study focused on a well-defined collection of 329 *S*. *aureus* isolates originating from animals in France and sampled over a 10-year period. Although geographically restricted to a single country and lacking the Bayesian inference required to formally estimate the timing of plasmid emergence or disappearance, this data set nevertheless provided valuable temporal insights into the local dynamics of plasmid dissemination, including their appearance, spread, and loss across animal-associated *S. aureus* isolates. Within this framework, a large diversity of ARGs was observed, the majority of them conferring resistances to β-lactams, aminoglycosides, and tetracyclines. This finding aligns with what has been observed in the large-scale study based on NCBI data (4) and is consistent with the fact that these three antibiotic classes have been the ones most commonly used for treating animals in France over the past decade (46). We further demonstrated that, in *S. aureus*, certain ARGs were consistently plasmid-borne, such as *str*, *erm*(C), *tet*(K), *tet*(L), and *cat(pC221*), while others were only occasionally carried on plasmids (e.g., *blaZ* and *aac(6′)-aph(2*″)) or were predominantly chromosomal (e.g., *tet*(M), *dfrC*, and *mecA*).

Although rare, transfer events from the chromosome to plasmids may be hypothesized, as observed with *blaZ*-carrying *rep20* plasmids from ST612 equine isolates (e.g., 48219, 54205, and 54213), which have recently acquired the *aac(6′)-aph(2*″) gene. This aminoglycoside-resistance gene predominantly identified as chromosomal in our whole-sequenced genome and in NCBI data (4) may have been mobilized onto plasmids, thanks to the presence of two flanking IS*256* elements. This hypothesis was further supported by the fact that the *aac(6′)-aph(2*″) gene sequences found on either the chromosome or plasmids were identical, potentially indicating recent transfer events. Conversely, while a decrease in plasmid-borne *blaZ* and a concomitant increase in its chromosomal localization were observed over time, our data do not support recent transfers from plasmids to the chromosome. Indeed, the chromosomal *blaZ* variant differed in sequence coverage and identity (94% for both) from the plasmid-borne version identified on the 27 kb *rep20* plasmid (e.g., 24506, 27262, and 32722). Sequence divergences reflect different evolutionary paths depending on the plasmid or chromosomal location (47), and the decrease in *blaZ* observed in animals in France is most probably due to modifications in the staphylococcal lineages circulating in the country.

The analysis of 211 plasmids from animal-associated *S. aureus* isolates from France revealed substantial diversity, yet three replicon families clearly dominated: *rep7a* (43%), *rep20* (19%), and *rep10* (13%). Strong associations between specific plasmid backbones and ARGs were observed, even though some genes were identified on two distinct plasmid families, namely, *erm*(C) on *rep10* and *repUS18*/*repUS12*, without any association with other mobile genetic elements, highlighting the remarkable ability of certain genes to disseminate efficiently on different genetic determinants (48). Each of the three major plasmid clusters was found across multiple STs and animal hosts, thereby supporting the hypothesis of horizontal plasmid transfer between diverse *S. aureus* lineages. Indeed, our analysis revealed that certain ARG/plasmid associations were not restricted to specific lineages but were found across multiple STs. While the spread of some plasmids reflected the clonal success of the ST (such as ST398 or ST8) by which they are harbored, others like *erm(C)/rep10* (found in six STs) or *str*/*rep7a* and *tet*(K)/*rep7a* (found in four STs) clearly indicated inter-ST dissemination. These plasmids, especially *rep7a* and *rep10* families, appeared to be efficient vectors for ARGs, contributing to their widespread presence in both livestock- and companion animal-associated isolates. The detection of identical plasmid profiles in unrelated STs and hosts further highlights the mobility and ecological versatility of plasmids, underscoring the role of plasmids as key drivers of resistance spread across genetic and host boundaries.

Ubiquitous small plasmids (<10 kb) comprised the majority of the *S. aureus* plasmidome—in line with previous reports (49–53)—whereas large plasmids (>20 kb) were relatively rare, representing less than a third of our collection. This distribution highlights the significant role of small plasmids in the ecology and evolution of *S. aureus* likely driven by rapid replication and unexpected transfer efficiency despite the absence of dedicated conjugation machinery (19). Among the small plasmids commonly observed in *S. aureus*, the *rep10* family (cluster C) stands out as the most conserved. This plasmid family is consistently associated with the *erm*(C) gene, which is invariably linked to a replicase encoded by the *rep10* gene, as already suggested in a large-scale study of over 10,000 genomes of *S. aureus* (4). These plasmids have been repeatedly reported not only in *S. aureus* (19, 51, 52, 54) but also, albeit less frequently, in other Firmicutes, such as *S. epidermidis* (18) and *Enterococcus faecalis* (55). Despite lacking canonical mobilization genes, *rep10* plasmids have been shown to transfer via transformation or transduction between *Staphylococcus* species and even across genera (56–58). These alternative mechanisms may account for their broad distribution across diverse STs and host origins.

This study also revealed the prevalence of *rep7a* plasmids (cluster B), which, despite their small size, were associated with multiple ARGs and harbored conserved backbones encoding mobilization functions, suggesting that they may act as mobilizable elements. These plasmids were frequently detected in strains also carrying larger plasmids—most commonly *rep20* family members. They have been previously described in *S. aureus* (19) and are commonly referred to by three prototypic names in the literature: (i) pT181, which carries the *tet(K*) gene, and a MOBV relaxase (59, 60), which corresponds to plasmids of the B2 group in our study; (ii) pC221, which displays the *cat*(*pC221*) gene, a replication gene, and several genes of unknown function (54) and is similar to p24614-2 (B3 group); and (iii) pS194, which harbors the *str* gene and a relaxase (61) and resembles p32084-1 (B7 group). In our study, temporal analysis of *rep7a* plasmids further revealed a significant evolution in both the *S. aureus* plasmidome and its associated resistome. Interestingly, the *rep7a* plasmid carrying both *cat(pC221*) and *str* (e.g., p50407 from B7 group) emerged in equine isolates, particularly in the Paris region and surrounding areas. In contrast, other *rep7a* plasmid variants, as well as *rep10* and *rep20* families, showed no apparent geographical clustering and were distributed more broadly across France. The high sequence similarity between *rep7a*/*cat(pC221)* (e.g., p32970-1, B13 group) and *rep7a*/*str* (e.g., 32084-1, B7 group) plasmids coupled with their rolling-circle replication may have facilitated recombination events leading to the formation of multi-resistance plasmids. Recombination has already been described as a key driver of plasmid evolution and adaptation, particularly in Gram-negative bacteria (62–64).

Apart from small and highly recombinant plasmids, we also observed significant genetic plasticity among larger plasmids (20–32 kb in size), particularly within the *rep20* plasmid family (cluster A), which exhibited marked heterogeneity despite being primarily associated with the *blaZ* gene. These plasmids were identified not only in livestock-associated *S. aureus* like ST398, but also in human-associated STs, such as ST8 and ST5, that were collected from cats and dogs. In the latter ST, *rep20* plasmids often appeared as hybrid plasmids frequently carrying an additional replicon, such as *rep5a* (49). The most abundant group within cluster A, A1, carried only *blaZ* and shared 99.9% identity with the 27 kb plasmid p19321-P03 from strain 19321, a community-associated MRSA isolate (65). It also closely resembled pSK67, an *S. aureus* plasmid historically reported in Australian hospitals between 1946 and 1981 (66). In addition to ARGs, these plasmids also harbored enterotoxin genes (*ser*, *sej*, *sep*) commonly associated with food-borne *S. aureus* outbreaks (67, 68). In this study, group A1 was exclusively detected in companion animal isolates (dogs, cats, and one rabbit) primarily belonging to ST8, suggesting possible human-to-animal transmission through close contact.

Our results showed that a single isolate often carried more than one plasmid, usually one large (mainly *rep20*, cluster A) and one small (often from the *rep10* or *rep7a* plasmids of clusters C and B, respectively). This pattern has also been observed in human-derived MRSA isolates in Malaysia, where large plasmids were found to coexist with *rep10* plasmids within the same genome (49). These findings are further supported by a large-scale analysis of *S. aureus* genomes from the NCBI database, which highlighted the frequent co-occurrence of multiple plasmid types (4). A few studies explicitly reported the coexistence of a large plasmid alongside a smaller one. However, large conjugative plasmids were capable of mobilizing smaller nonconjugative ones in *S. aureus* (5, 69). Although only one conjugative plasmid was formally identified in our data set, this absence likely reflects limitations in current bioinformatic tools rather than a true absence of such elements. Moreover, the coexistence of multiple plasmids within a single isolate facilitates the formation of hybrid plasmids, which have been reported in previous studies (70). For example, in plasmids from the D8 group, the association of *repUS12* and *repUS18* was linked with *aadD*, *erm*(C), and *tet*(L) genes. The *rep20* replicon was also found in association with *rep21* in hybrid plasmids belonging to the A4 group, as previously described (71). The *rep 21* gene encodes a Rep1-type replication protein typically found in small cryptic plasmids (<3 kb), which have been identified in clinical MRSA human isolates (49) and are prone to integration into larger plasmids (~30 kb).

Here, *rep20*/*rep21* plasmids closely resembled 24 kb A4 group plasmids carrying only *rep20* and *blaZ*, with the only differences being in the insertion of *rep21* near an origin of transfer and the IS*Sau6* element, along with the insertion of the *qac* gene (e.g., p33658). This configuration suggests the integration of a small Rep1*-*type plasmid into a larger backbone. The Rep1 domain of replication proteins has been shown to act as dual-function replication-relaxase proteins supporting the mobilization of otherwise nonconjugative plasmids (72, 73). Lee et al*.* (72) demonstrated that in *Bacillus subtilis*, ICE elements can mediate the transfer of Rep1-type plasmids through a relaxase-independent mechanism (72). In our study, relaxase activity was also predicted in certain *repUS18*/*repUS12* plasmids that encode replication proteins with Inc18 and Rep1 domains, respectively, and were found to carry *aadD*, *tet*(L), and *erm*(C) genes. The presence of such mosaic plasmids comprising multiple replicons and ARGs underscores the remarkable plasticity and adaptive potential of *S. aureus* plasmidome.

Finally, the remarkable plasticity of *S. aureus* plasmids was further evidenced by the identification of an atypical mosaic plasmid, p33622-1, composed of backbone elements originating from both *S. aureus* and *Enterococcus faecium*. Although the *cfr* gene commonly associated with resistance to linezolid was not present in the plasmids identified in our isolates, this hybrid structure suggests that plasmid-mediated exchange of the *cfr* gene between species is possible. Supporting this, a previous study demonstrated the successful transfer of a *cfr*-carrying plasmid from *Enterococcus* to *S. aureus* (74). Recombination between these two plasmids may have occurred through the IS shared by both plasmids, facilitating inter-species genetic exchange. These findings further confirm the broad host range and dynamic nature of *S. aureus* plasmids, which can expand their reservoir of ARGs through horizontal gene transfer across species. Such mosaic plasmids enhance the genetic diversity and evolutionary potential of *S. aureus*, emphasizing the importance of understanding plasmid mobility and recombination in the context of antimicrobial resistance dissemination.

### Conclusion

This study demonstrated the significant diversity of plasmids in *S. aureus* isolates of animal origin, with the predominance of the *rep7a*, *rep20*, and *rep10* families. Despite their relatively small size*,* these plasmids were widespread and driven by their genetic plasticity, broad host range, and capacity for mobilization and homologous recombination.

While plasmid dissemination in *S. aureus* has traditionally been linked to epidemic clones, our results suggest that plasmids are also spreading horizontally. This was highlighted in this longitudinal analysis by the detection of specific plasmids, including small multi-resistance ones, in different STs. Overall, our analyzes underscored the notion that plasmids most likely act as dynamic vehicles for ARGs in *S. aureus*. These findings highlight the critical need to unravel the mechanisms driving the epidemic success and widespread dissemination of ARG-carrying plasmids in *S. aureus*.

## Data Availability

All plasmid and chromosomal raw data are available on the NCBI’s Sequence Read Archive (SRA) under BioProject PRJNA1193534.

## SUPPLEMENTAL MATERIAL

Figure S1 spectrum.00772-25-s0001.pdf PFGE profiles.

Supplemental material spectrum.00772-25-s0002.docx Supplemental methods, Tables S2 and S3, and Fig. S2 to S6.

Table S1 spectrum.00772-25-s0003.xlsx List of strains and their metadata.

Table S4 spectrum.00772-25-s0004.xlsx Quality control of the 81 sequenced *S. aureus* genomes.

Table S5 spectrum.00772-25-s0005.xlsx List of plasmids and their metadata.

ASM does not own the copyrights to Supplemental Material that may be linked to, or accessed through, an article. The authors have granted ASM a non-exclusive, world-wide license to publish the Supplemental Material files. Please contact the corresponding author directly for reuse.
