# Supplementary material for: Comprehensive genomic analysis of antibiotic resistance plasmids in animal-associated Staphylococcus aureus in France
Source: Microbiol Spectr. 2025 Sep 18;13(10):e00772-25. doi: 10.1128/spectrum.00772-25 (PMC12502713; doi:10.1128/spectrum.00772-25)
Supplement: Figure S1 — PFGE profiles. [file spectrum.00772-25-s0001.pdf]

A

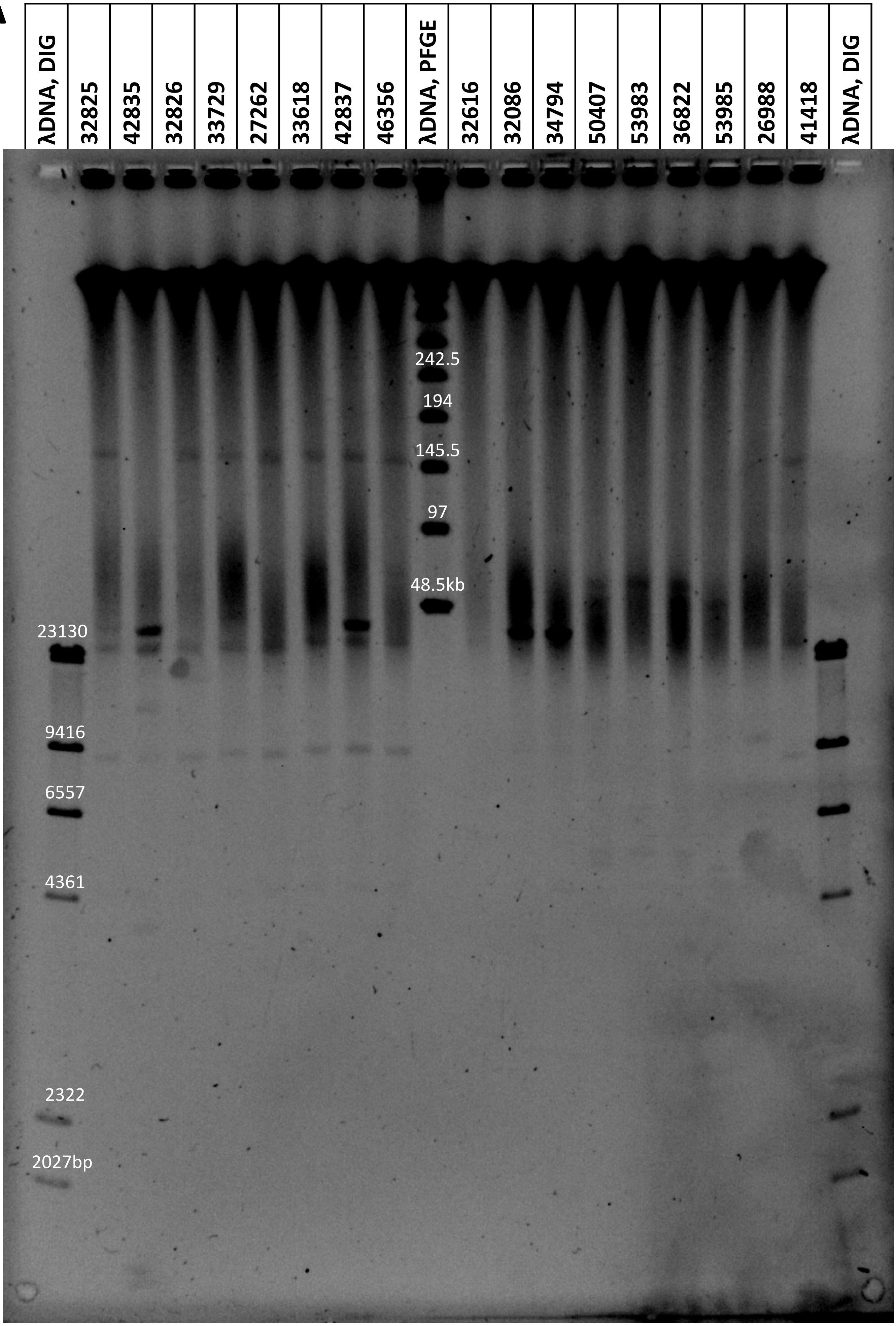
λDNA, DIG
32825
42835
32826
33729
27262
33618
42837
46356
λDNA, PFGE
32616
32086
34794
50407
53983
36822
53985
26988
41418
λDNA, DIG
242.5
194
145.5
97
48.5kb
23130
9416
6557
4361
2322
2027bp

B

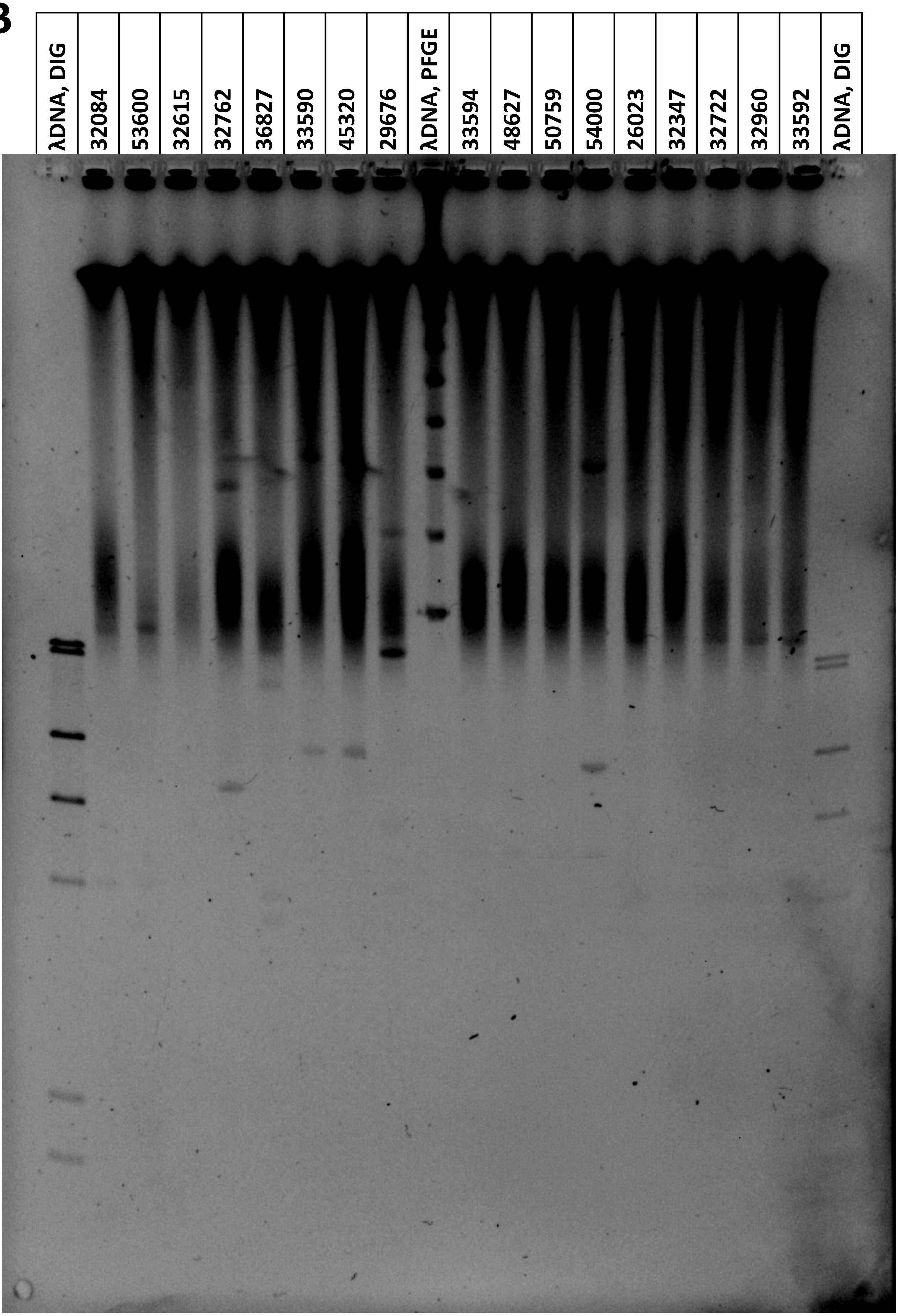

λDNA, DIG
32084
53600
32615
32762
36827
33590
45320
29676
λDNA, PFGE
33594
48627
50759
54000
26023
32347
32722
32960
33592
λDNA, DIG

C

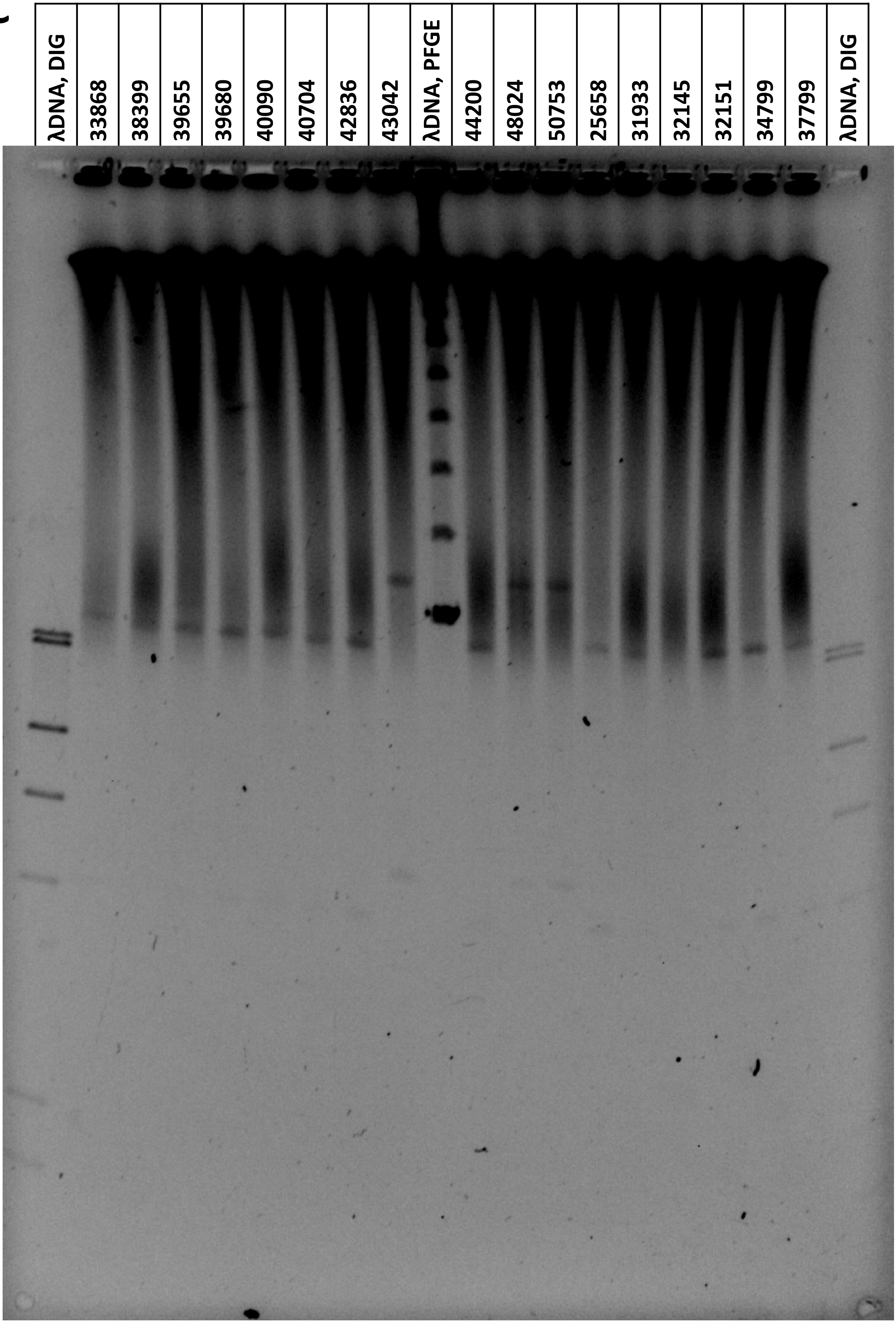
λDNA, DIG
33868
38399
39655
39680
40090
40704
42836
43042
λDNA, PFGE
44200
48024
50753
25658
31933
32145
32151
34799
37799
λDNA, DIG

D

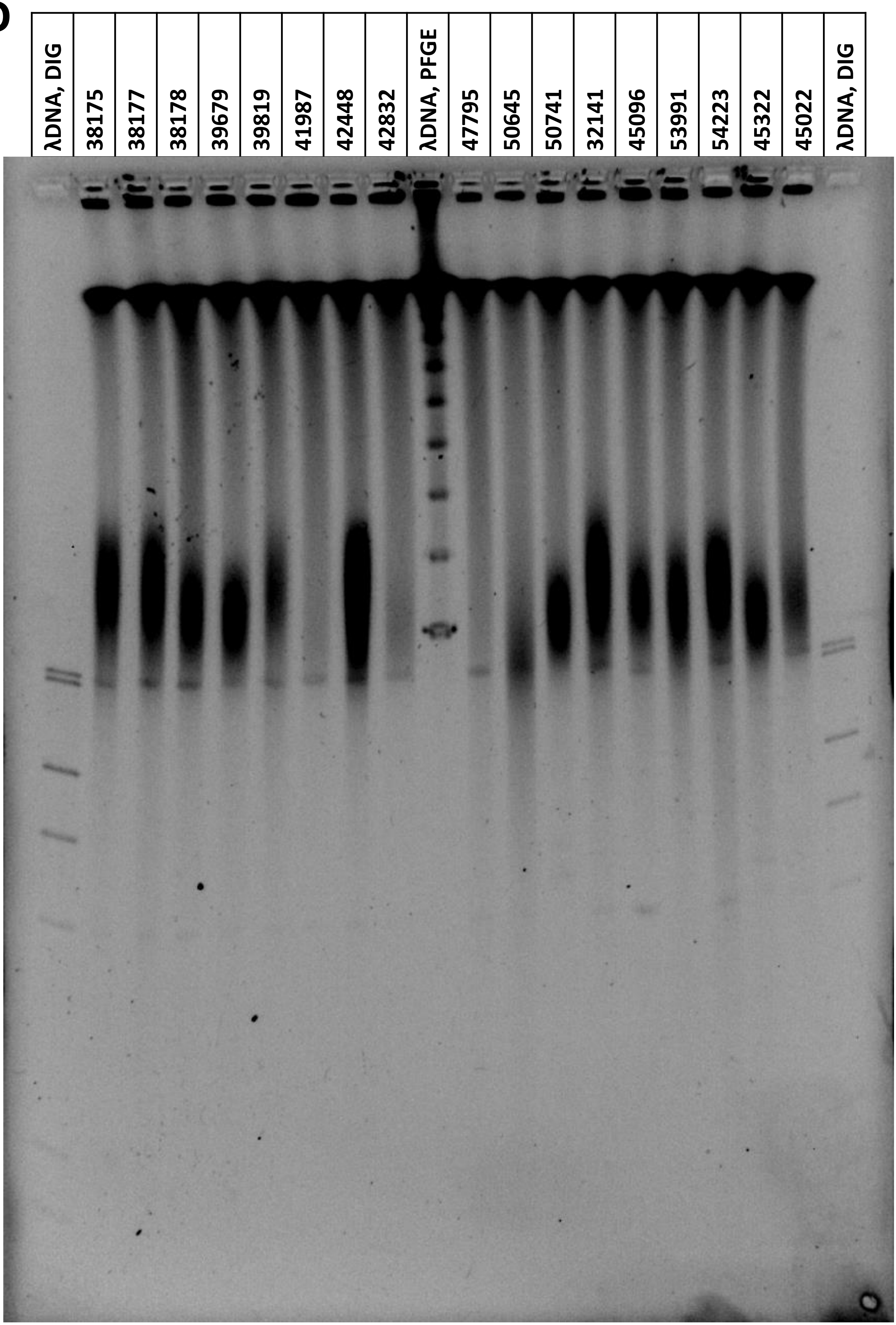
λDNA, DIG
38175
38177
38178
39679
39819
41987
42448
42832
λDNA, PFGE
47795
50645
50741
32141
45096
53991
54223
45322
45022
λDNA, DIG

E

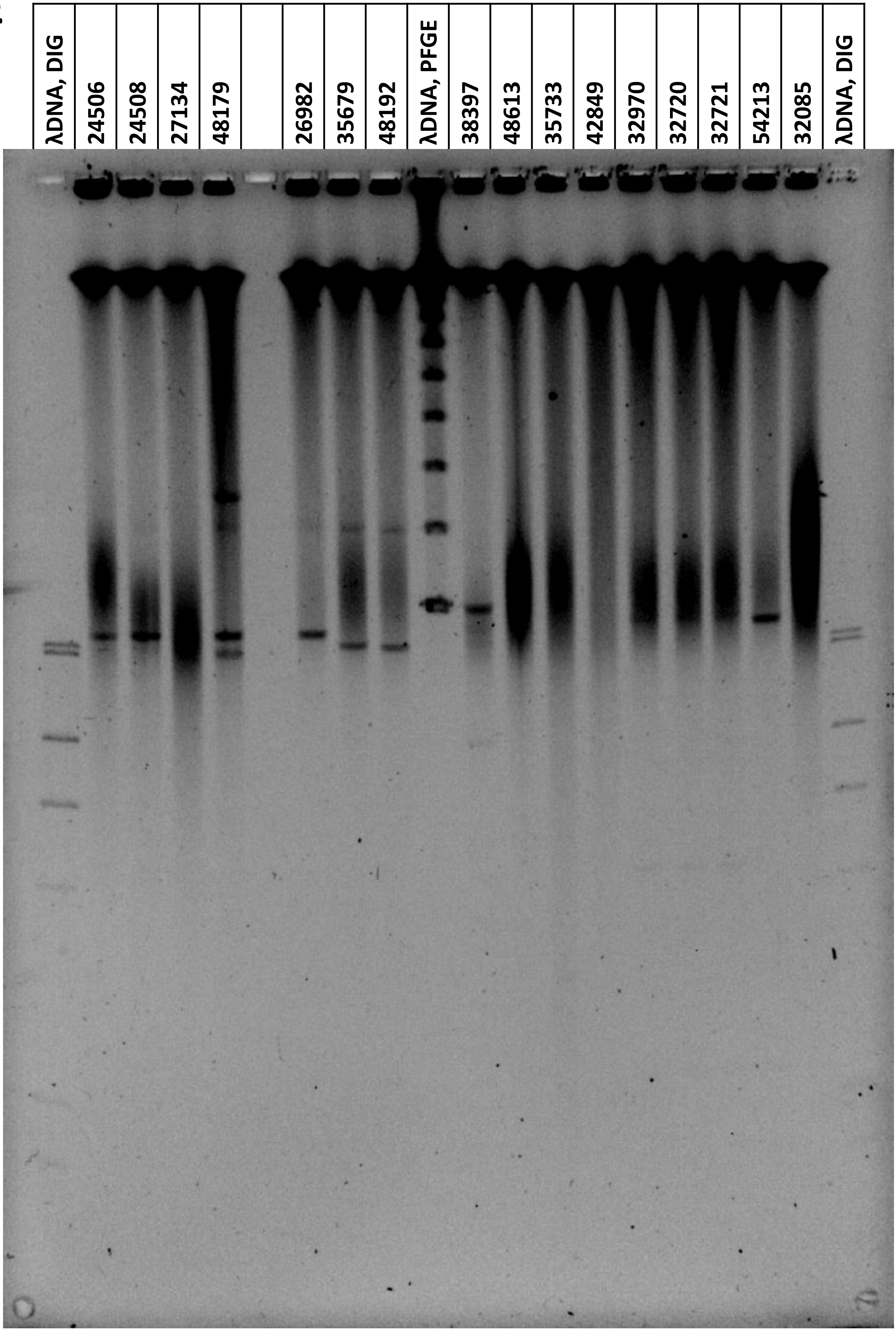
λDNA, DIG
24506
24508
27134
48179
26982
35679
48192
λDNA, PFGE
38397
48613
35733
42849
32970
32720
32721
54213
32085
λDNA, DIG

F

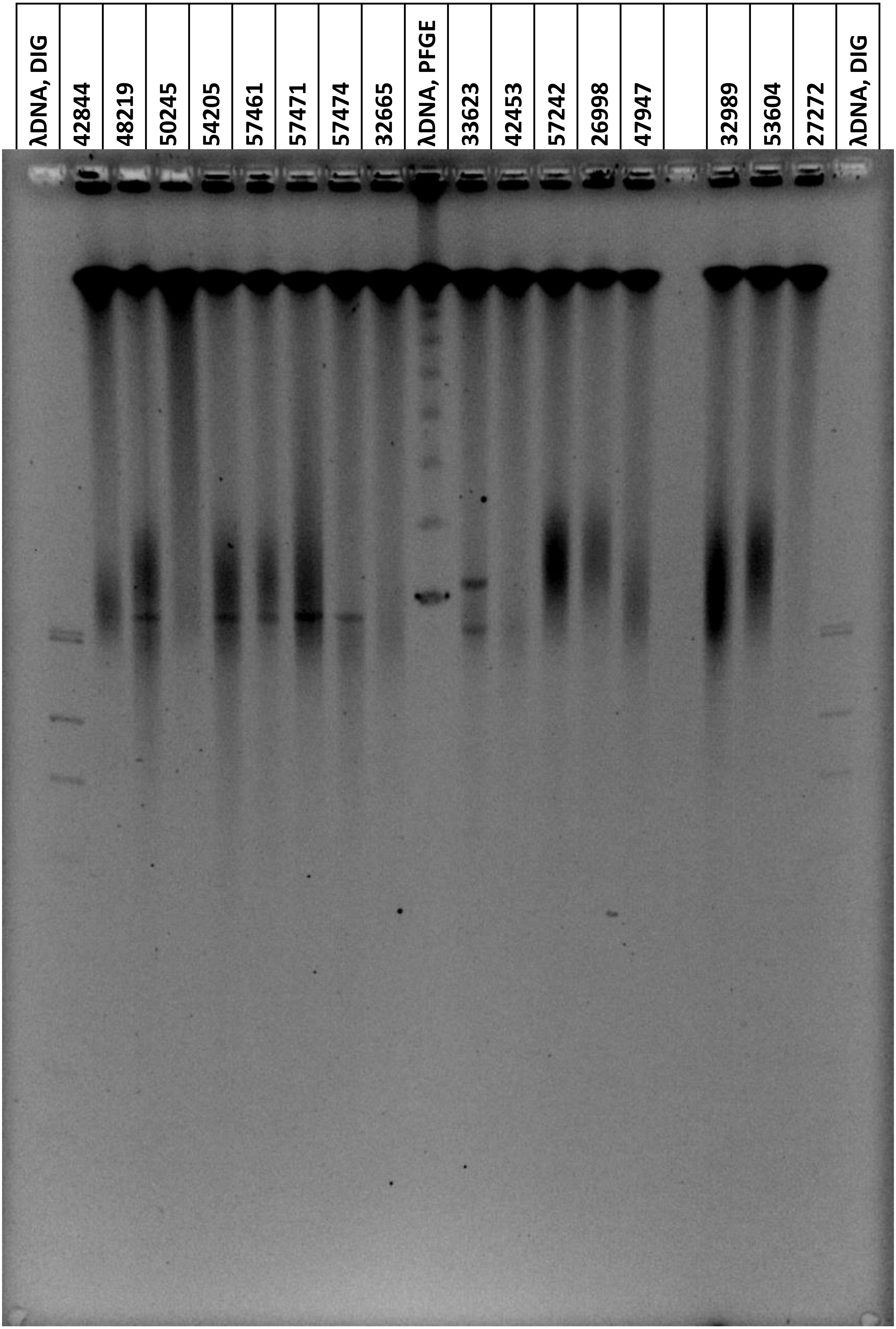
λDNA, DIG
42844
48219
50245
54205
57461
57471
57474
32665
λDNA, PFGE
33623
42453
57242
26998
47947
32989
53604
27272
λDNA, DIG

G

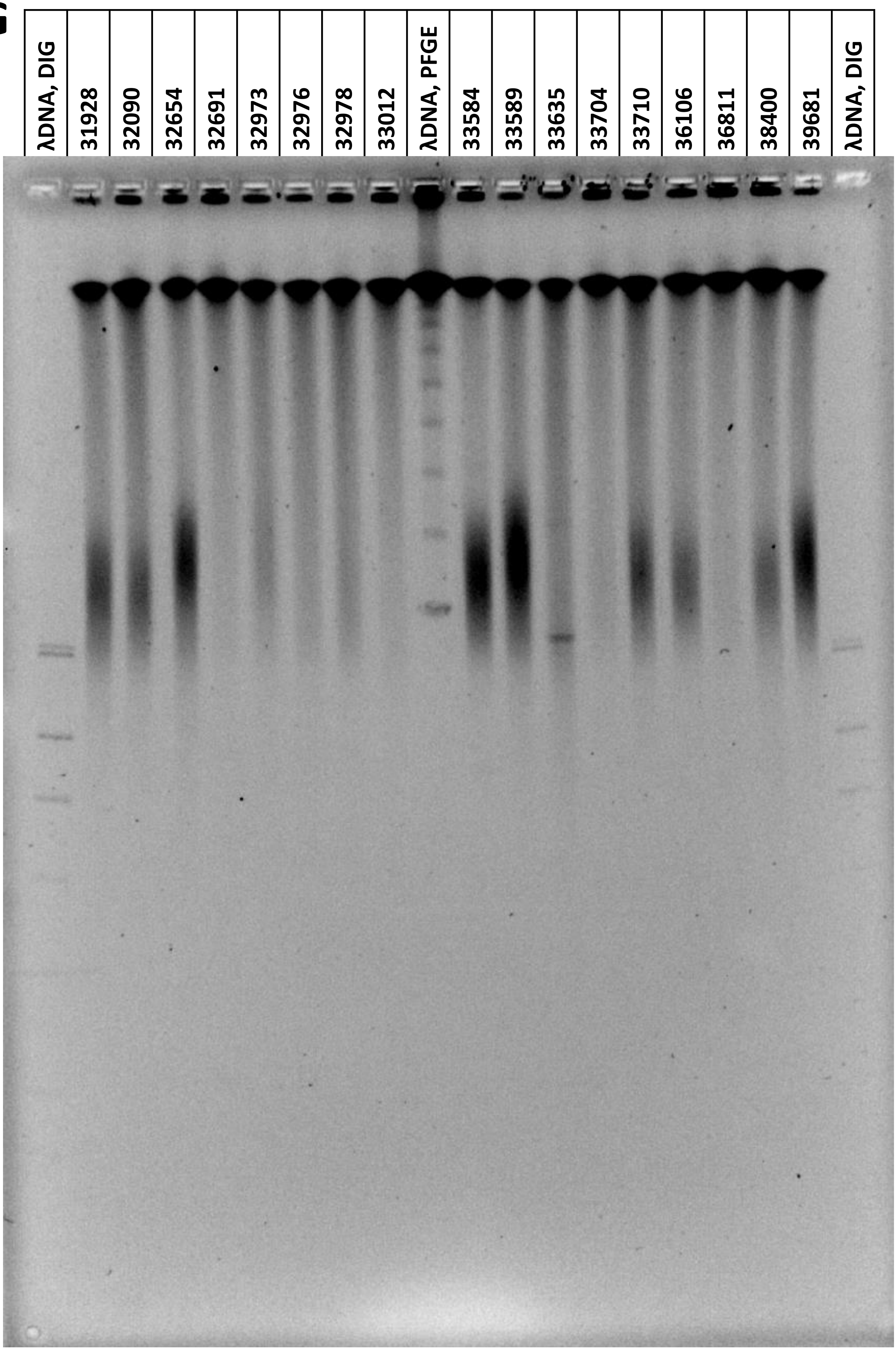
λDNA, DIG
31928
32090
32654
32691
32973
32976
32978
33012
λDNA, PFGE
33584
33589
33635
33704
33710
36106
36811
38400
39681
λDNA, DIG

H

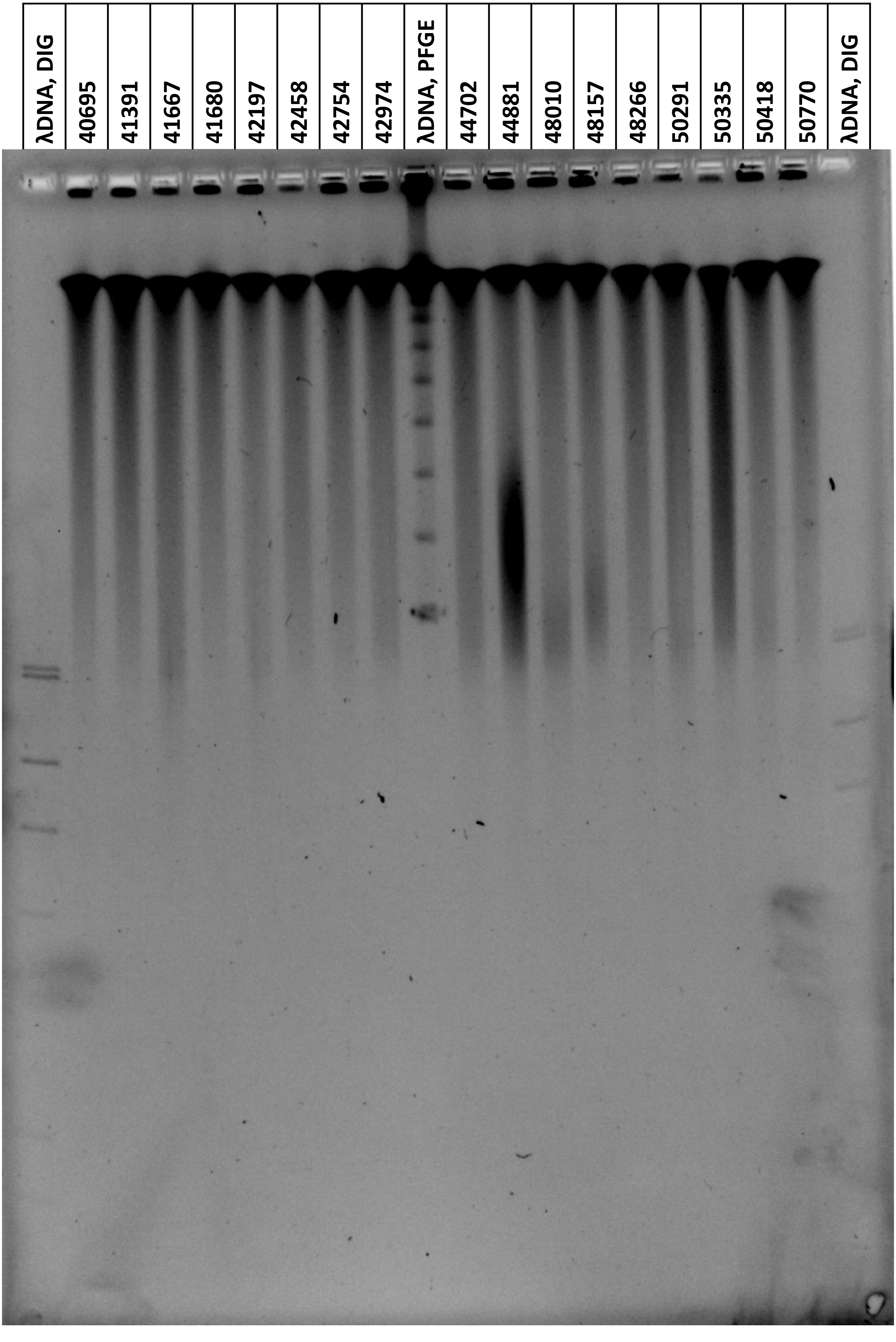

λDNA, DIG
40695
41391
41667
41680
42197
42458
42754
42974
λDNA, PFGE
44702
44881
48010
48157
48266
50291
50335
50418
50770
λDNA, DIG

I

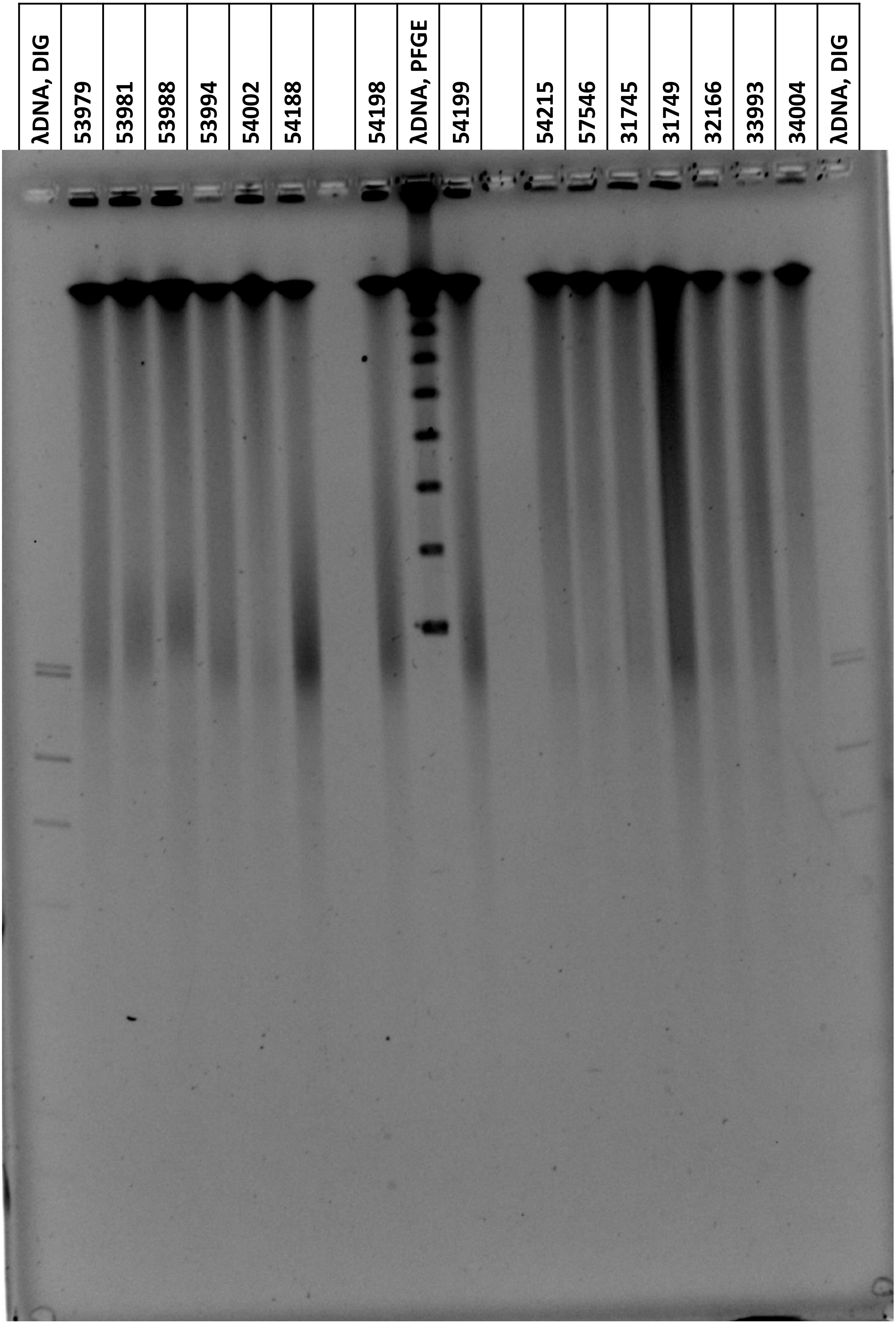
λDNA, DIG
53979
53981
53988
53994
54002
54188
54198
λDNA, PFGE
54199
54215
57546
31745
31749
32166
33993
34004
λDNA, DIG

J

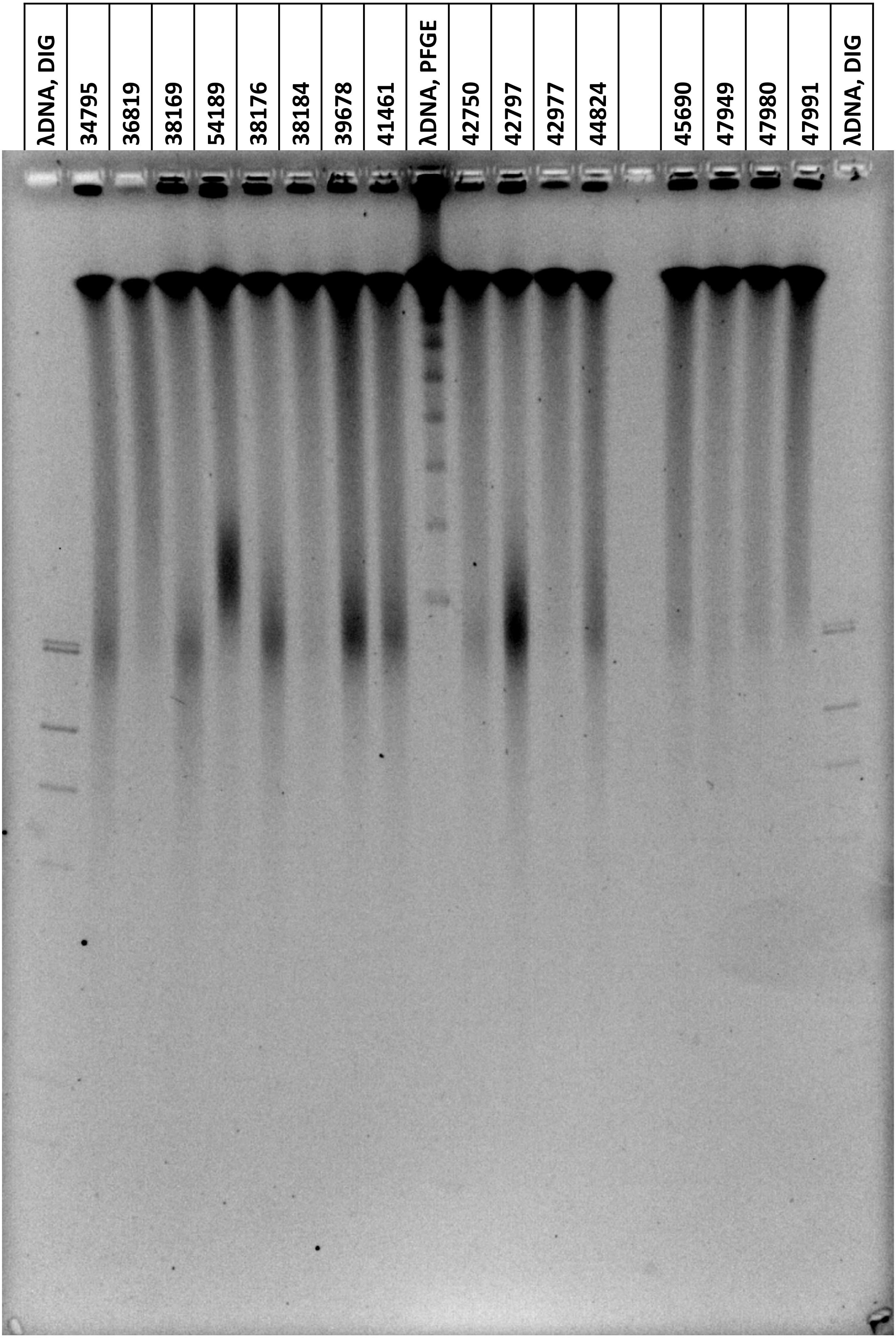
λDNA, DIG
34795
36819
38169
54189
38176
38184
39678
41461
λDNA, PFGE
42750
42797
42977
44824
45690
47949
47980
47991
λDNA, DIG

K

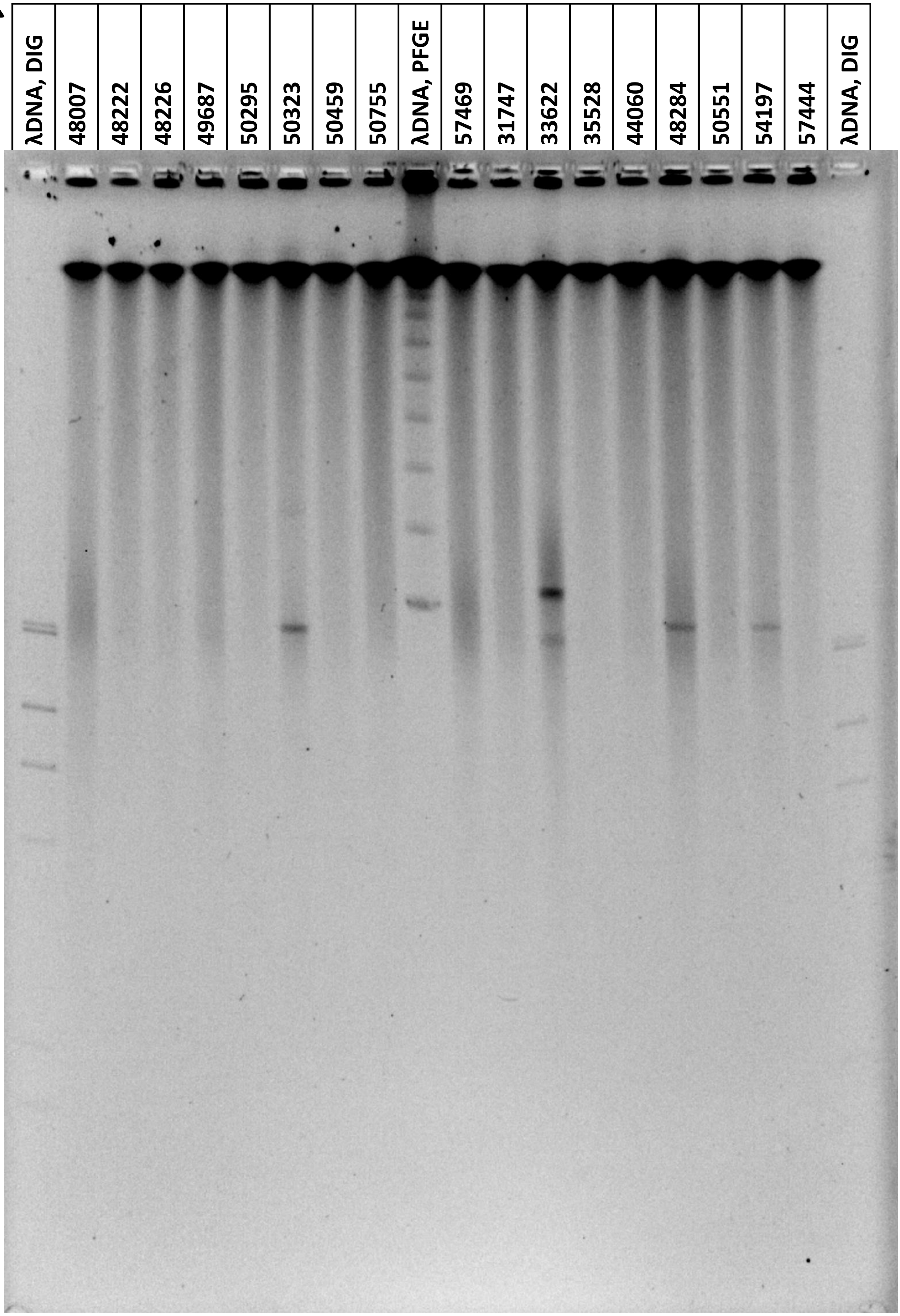
λDNA, DIG
48007
48222
48226
49687
50295
50323
50459
50755
λDNA, PFGE
57469
31747
33622
35528
44060
48284
50551
54197
57444
λDNA, DIG

L

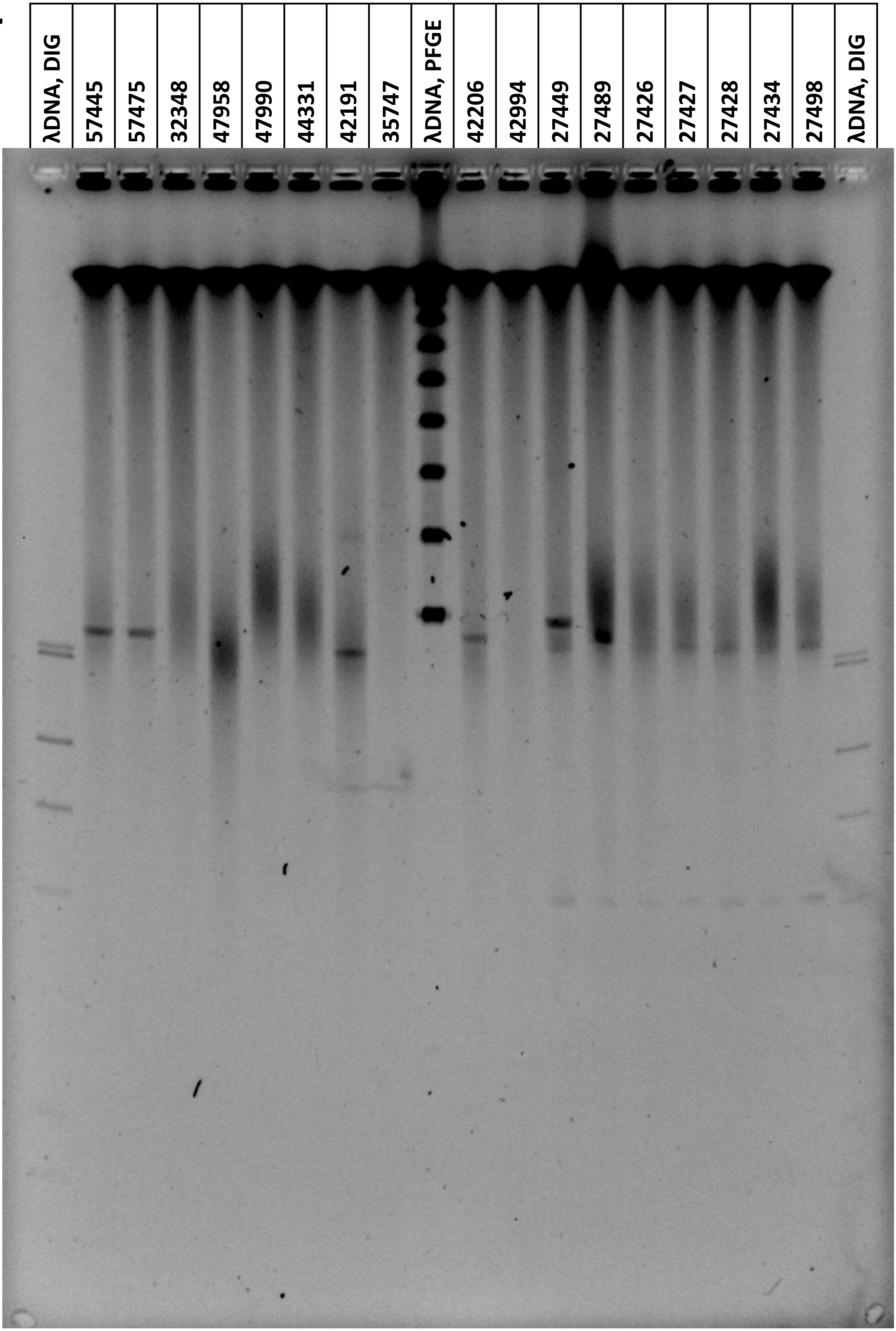
λDNA, DIG
57445
57475
32348
47958
47990
44331
42191
35747
λDNA, PFGE
42206
42994
27449
27489
27426
27427
27428
27434
27498
λDNA, DIG

M

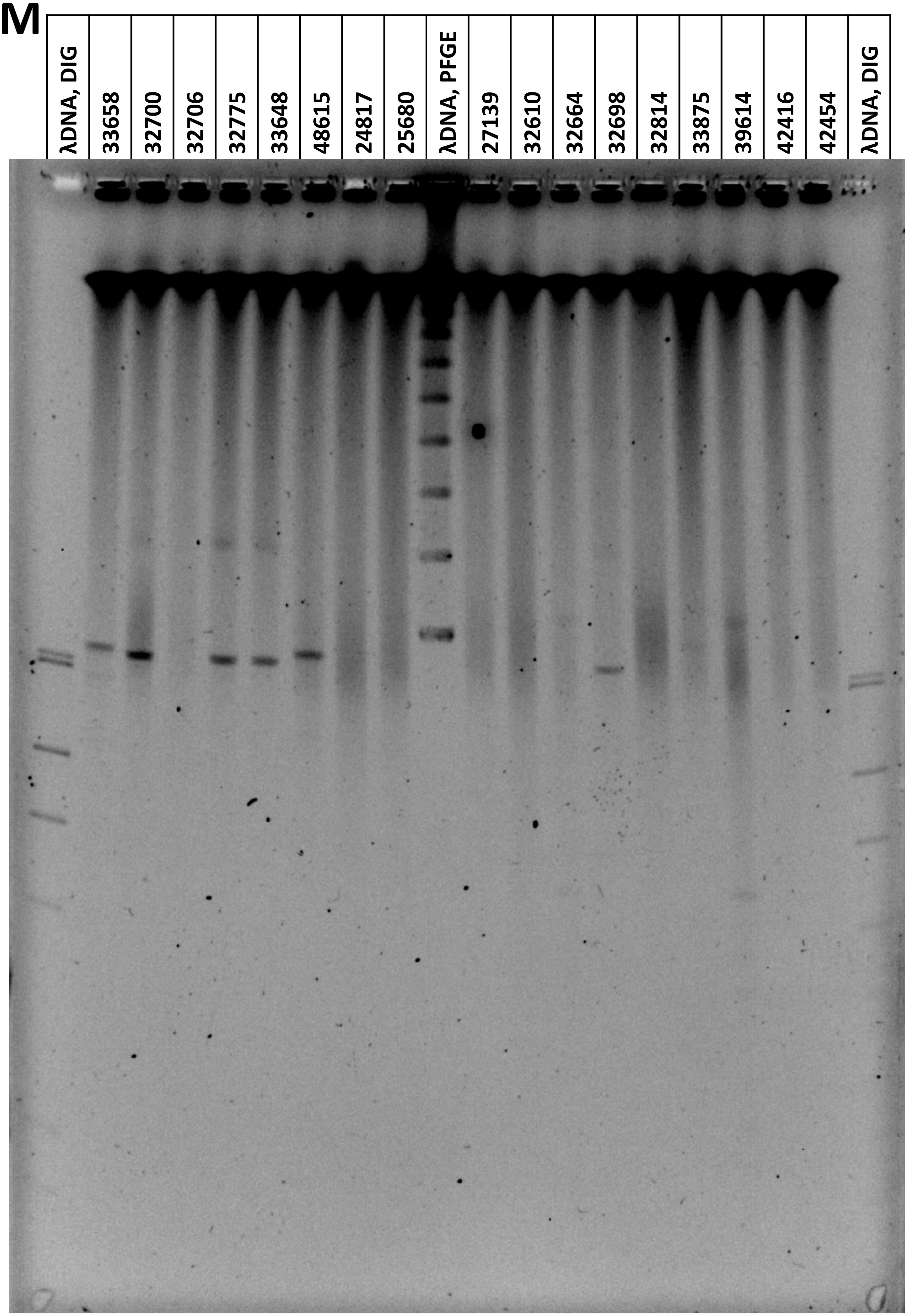

λDNA, DIG
33658
32700
32706
32775
33648
48615
24817
25680
λDNA, PFGE
27139
32610
32664
32698
32814
33875
39614
42416
42454
λDNA, DIG

N

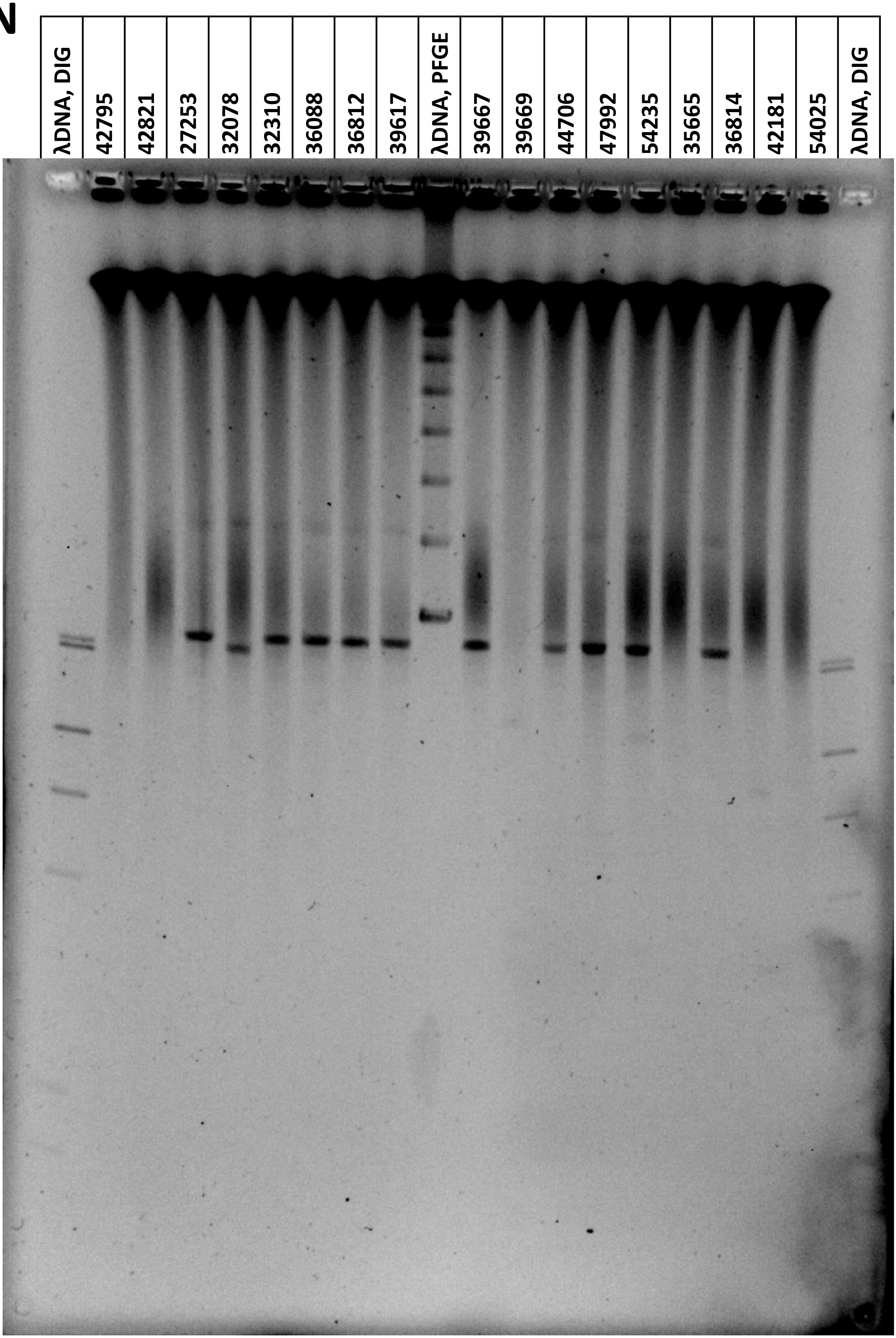
λDNA, DIG
42795
42821
27253
32078
32310
36088
36812
39617
λDNA, PFGE
39667
39669
44706
47992
54235
35665
36814
42181
54025
λDNA, DIG

O

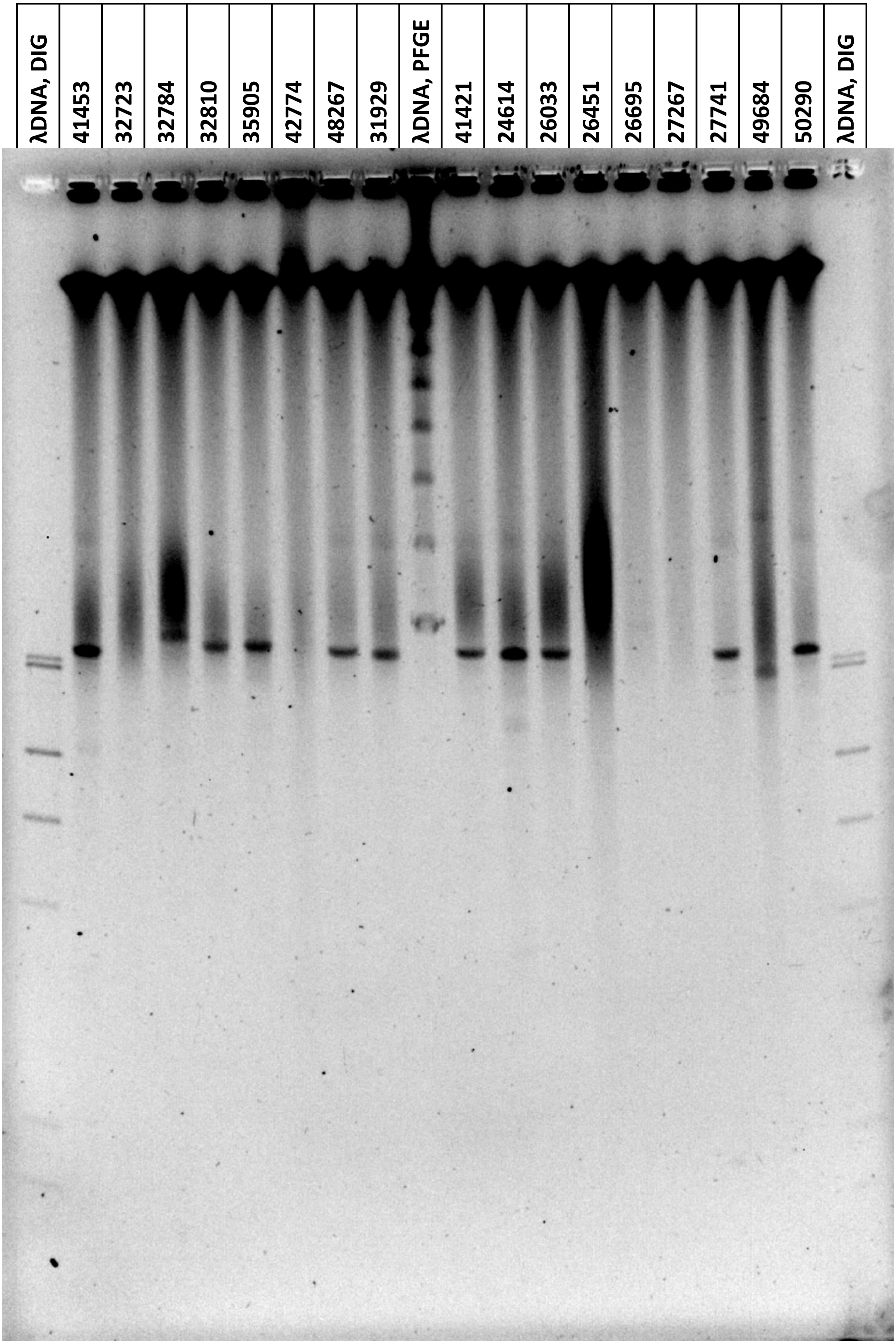
λDNA, DIG
41453
32723
32784
32810
35905
42774
48267
31929
λDNA, PFGE
41421
24614
26033
26451
26695
27267
27741
49684
50290
λDNA, DIG

P

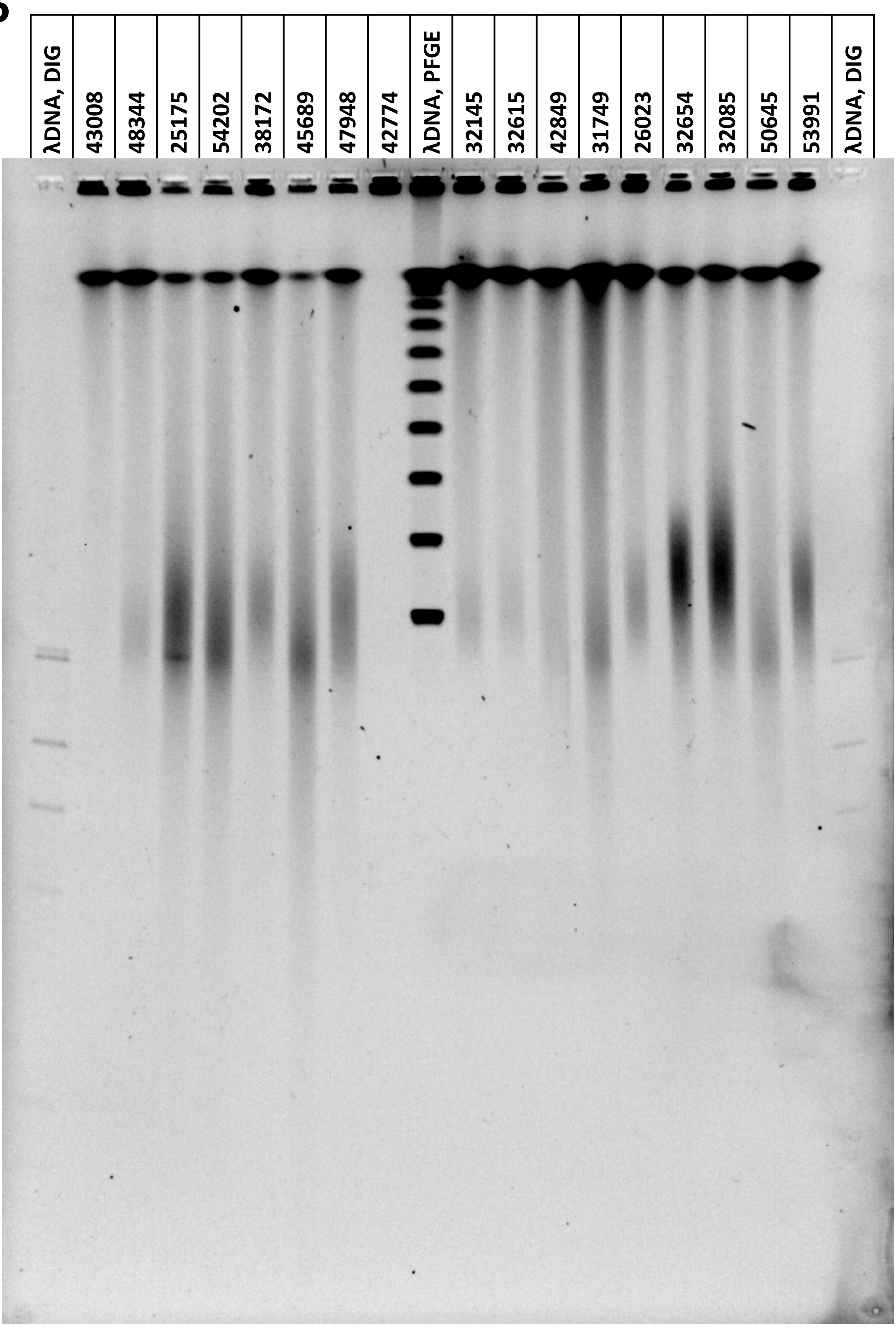

λDNA, DIG
43008
48344
25175
54202
38172
45689
47948
42774
λDNA, PFGE
32145
32615
42849
31749
26023
32654
32085
50645
53991
λDNA, DIG

Figure S1.
See Supplementary_data.docx for the legend
