# Supplementary material for: Comprehensive genomic analysis of antibiotic resistance plasmids in animal-associated Staphylococcus aureus in France
Source: Microbiol Spectr. 2025 Sep 18;13(10):e00772-25. doi: 10.1128/spectrum.00772-25 (PMC12502713; doi:10.1128/spectrum.00772-25)
Supplement: Supplemental material — Supplemental methods, Tables S2 and S3, and Fig. S2 to S6. [file spectrum.00772-25-s0002.docx]

**Supplementary material**

**Pulsed-field gel electrophoresis**

*S. aureus* isolates were grown on COS medium overnight at 37 °C, and 5 mL of BHI medium was inoculated with the overnight bacterial culture. The culture was incubated at 37 °C under shaking until it ran OD_600nm_=1. A 1 mL culture volume was then centrifuged for ten minutes at 5000 rpm. The pellet was resuspended in 200 µL of TEE buffer (10 mM Tris, 100 mM EDTA, pH 9). A 50 µL volume was mixed with 50 µL of preheated 2% CleanCut agarose. For bacterial lysis, 8 µL of lysostaphin (0.5 mg/mL) was added to the mixture. To form the plugs, 23 µL of the bacteria/agarose mixture was pipetted as a drop onto a plate until it polymerized. For bacterial lysis, the plugs were placed in a tube containing 1 mL of ST lysis buffer (6 mM Tris-HCl, pH 8; 1 M NaCl; 0.1 M EDTA; 0.2% sodium deoxycholate; and 0.5% N-lauroylsarcosine), 10 µL of lysozyme (100 mg/mL), and 50 µL of 10% Brij 58. The plugs with this solution were incubated for two hours at 300 rpm at 37 °C. For protein lysis, the solution was replaced by 1 mL of ES buffer (0.5 M EDTA, 1% N-lauroylsarcosine, pH 8) with 1 mg/mL proteinase K. The mixture was then incubated at 55 °C under shaking at 300 rpm for 18 hours. After cooling to room temperature, the plugs were washed by replacing the previous buffer with TE buffer (10 mM Tris-HCl, pH 8, and 1 mM EDTA) and agitated at 300 rpm at room temperature for 30–45 minutes. This washing step was repeated four or five times. Then, plugs were incubated with 300 µL of 0.1% Triton X-114 for one hour at 4 °C. The Triton was removed and immediately replaced by TE buffer. To linearize plasmids, the DNA was digested with the S1 nuclease (Promega). Plugs were incubated with 80 µL of digestion buffer (72 µL of water, 8 µL of S1 buffer, and 0.2 µL of S1 nuclease) for 30 to 45 minutes at 37 °C under shaking at 300 rpm, and then washed twice with TE buffer. Plugs and a size marker (CHEF DNA Size Standard, 48.5–1,000 kb Lambda Ladder, BioRad) were run in 1% Certified Megabase agarose gels in 0.5X TBE buffer. The migration was performed using the following parameters: 16 hours of migration, a gradient of 6 V/cm, an initial switch of 1 s, a final switch of 25 s, a 120° angle, linear. Gels were stained using ethidium bromide and visualized under UV light.

**Supplementary tables and figures**

**Table S1: Database of phenotype resistance, antibiotic resistance genes (ARG), and mobile genetic elements (MGE) detected in the 329 *S. aureus* isolates from the Resapath network and analyzed in this study. (see *TableS1.xlsx* file)**

**Table S2:** **Primers used in this study to detect antibiotic resistance genes in *S. aureus* isolates.**

**Table S3: Primers used to design probes for dot blot.**

**Table S4: Quality control of the 81 sequenced *S. aureus* genomes. (see *TableS4.xlsx* file)**

**Table S5: Database of plasmids detected in 139 *S. aureus* isolates, their antibiotic resistance genes (ARGs), and associated mobile genetic elements (MGEs). (see *TableS5.xlsx* file)**

**Figure S1: Pulsed-field gel electrophoresis (PFGE) profiles showing the presence of plasmids in *S. aureus* strains.** For all gels (A–O), the names of the strains are indicated above each lane. Two molecular weight markers were used: the first, labeled “λDNA, DIG,” corresponds to the DNA Molecular Weight Marker II, DIG-labeled (Roche); the second, labeled “λDNA, PFGE,” is the CHEF DNA Size Standard Lambda Ladder (Bio-Rad). Each band observed, except for the upper smear corresponding to chromosomal DNA, corresponds to a plasmid. The sizes of the different bands of the two markers are indicated on the first gel (A). **O.** Thiourea was added to the TBE buffer during the revelation step. *(see Figure_S1.pdf file)*

**Figure S2: Occurrence of antibiotic resistance genes (ARGs) in *S. aureus* based on their host.** *S. aureus* hosts are listed on the left-hand side of the heatmap, detected ARGs at the bottom, and the total number of genomes analyzed for each host on the right-hand side. The percentages of genomes containing ARGs were calculated by dividing the number of genomes carrying an ARG by the total number of genomes, as displayed in the top right of the heatmap. These percentages are represented by different shades of red, as indicated in the color scale at the bottom of the heatmap.

**Figure S3: Occurrence of *S. aureus* resistant to an antibiotic family (A) or a specific antibiotic (B) according to their host.** *S. aureus* hosts were listed on the left-hand side of the heatmap, with antibiotic families (A) and specific antibiotics (B) at the bottom. (A) The percentages of isolates with a resistance phenotype were calculated by dividing the number of isolates resistant to at least one antibiotic in the family by the total number of isolates. (B) The percentages of isolates resistant to specific antibiotics were calculated by dividing the number of resistant isolates by the total number of isolates. These percentages are represented by different shades of red, as indicated in the color scale at the bottom of the heatmap.

**Figure S4: Sizes of plasmids in animal-derived *S. aureus* strains.** The different plasmid size ranges isolated from *S. aureus* strains are indicated on the right-hand side in kb. In the pie chart, the number of plasmids identified for each size range is shown at the end of each sector.

**Figure S5: Map of the large atypical plasmids of *S. aureus* isolates** **42837 (A) and 33622 (B).** Plasmid annotation was performed using Bakta ([Schwengers *et al.*, 2021](https://pubmed.ncbi.nlm.nih.gov/34739369/)), and visualization was done with SnapGene (<https://www.snapgene.com/>). Coding sequences with similar functions are color-coded according to the legend in the bottom right. The name and size of each plasmid are indicated at the center of each sequence. Genes of interest were also annoted in the figure.

**Figure S6: Evolution of occurrences of five resistance genes and their associations with plasmids over time in *S. aureus* isolates.** The genes *cat(pC221)* and *str* are shown in A, *aadD* and *tet(L)* in B, and the *blaZ* gene in C. The percentage per year of isolates carrying each gene is represented by a curve, calculated by dividing the number of identified genes (whether on the chromosome or a plasmid) by the total number of isolates collected that year. The percentage of isolates carrying genes on plasmids is represented by a histogram, calculated by dividing the number of gene/plasmid associations found per year by the total number of isolates collected that year. Consequently, when the bar of the histogram is near the curve (as with *tet*(L)), this means that the gene is primarily plasmid-borne. In contrast, when the bar of the histogram is far below the curve (as with *blaZ* and *aadD*), this means that the gene is mostly located on the chromosome. Each gene and each association is represented by a distinct color, as indicated at the bottom of each chart.

**Table S2**

| **Multiplex PCR** | **Antibiotic resistance gene** | **Protein** | **Oligonucleotide name** | **Oligonucleotide sequence (5'-3')** | **Amplicon size (bp)** | **Reference** | **PMID** |
| --- | --- | --- | --- | --- | --- | --- | --- |
| Multiplex aminoglycoside | *aac(6')-aph (2'')* | Aminoglycoside acetyltransferase | Aac_F | CAGAGCCTTGGGAAGATGAAG | 348 | Khosravi *et al.*, 2017 | [29132547](https://pubmed.ncbi.nlm.nih.gov/29132547/) |
|  |  |  | Aac_R | CCTCGTGTAATTCATGTTCTGGC |  | Khosravi *et al.*, 2017 | [29132547](https://pubmed.ncbi.nlm.nih.gov/29132547/) |
|  | *aph(3')-III* | Aminoglycoside phosphotransferase | Aph3_F | GGCTAAAATGAGAATATCACCGG | 526 | Khosravi *et al.*, 2017 | [29132547](https://pubmed.ncbi.nlm.nih.gov/29132547/) |
|  |  |  | Aph3_R | CTTTAAAAAATCATACAGCTCGCG |  | Khosravi *et al.*, 2017 | [29132547](https://pubmed.ncbi.nlm.nih.gov/29132547/) |
|  | *aadD* | Aminoglycoside adenyltransferase | aadD-F1 | GAATATTGGATAAATATGGGGATGA | 664 | Fraiture *et al.*, 2020 | [32341433](https://pubmed.ncbi.nlm.nih.gov/32341433/) |
|  |  |  | aadD-R1 | TATCCGTGTCGTTCTGTCCA |  | Fraiture *et al.*, 2020 | [32341433](https://pubmed.ncbi.nlm.nih.gov/32341433/) |
|  | *str* (synonym *aadK****)*** | Aminoglycoside adenyltransferase | str-F | GAGGGTTCAAGAACTAATG | 432 | Hauschild *et al.*, 2007 | [17650957](https://pubmed.ncbi.nlm.nih.gov/17650957/) |
|  |  |  | str-R | AACACCCTTTGCTACATA |  | Hauschild *et al.*, 2007 | [17650957](https://pubmed.ncbi.nlm.nih.gov/17650957/) |
| Multiplex *ant6* and *ant9* | *ant(6)-Ia* | Aminoglycoside nucleotidyltransferase | aadE_fw | GCAGAACAGGATGAACGTATTCG | 553 | Li *et al.,* 2013 | [23386262](https://pubmed.ncbi.nlm.nih.gov/23386262/) |
|  |  |  | aadE_rv | TTATCCCAACCTTCCACGAC |  | Li *et al.,* 2013 | [23386262](https://pubmed.ncbi.nlm.nih.gov/23386262/) |
|  | *ant(9)-Ia* | Aminoglycoside nucleotidyltransferase | ant9_FOR | AGTGAAGTTGTCCCTTGGCA | 298 | This study |  |
|  |  |  | ant9_REV | GCCACATTCGAGCTAGGGTT |  | This study |  |
| Multiplex BVQ | *blaZ* | Class A beta-lactamases | BlaZ 1 | AAGAGATTTGCCTATGCTTC | 517 | Sawant *et al*., 2009 | [18950969](https://pubmed.ncbi.nlm.nih.gov/18950969/) |
|  |  |  | BlaZ 2 | GCTTGACCACTTTTATCAGC |  | Sawant *et al*., 2009 | [18950969](https://pubmed.ncbi.nlm.nih.gov/18950969/) |
|  | *vga(E)*** | ABC-transporter | vgaE_F6 | GAAATATGGGAAATAGAAGATGG | 995 | Li *et al.*, 2014 | [24324222](https://pubmed.ncbi.nlm.nih.gov/24324222/) |
|  |  |  | vgaE_R3 | TGATTCTCTAACCACTCTTC |  | Li *et al.*, 2014 | [24324222](https://pubmed.ncbi.nlm.nih.gov/24324222/) |
|  | *qacG* | efflux pump | qacG_for | CAACAGAAATAATCGGAACT | 275 | Bjorland *et al.,* 2005 | [16145078](https://pubmed.ncbi.nlm.nih.gov/16145078/) |
|  |  |  | qacG_rev | TACATTTAAGAGCACTACA |  | Bjorland *et al.,* 2005 | [16145078](https://pubmed.ncbi.nlm.nih.gov/16145078/) |
| Multiplex DQ | *qacA* | efflux pump | qacA_F | GCTGCATTTATGACAATGTTTG | 630 | Kong *et al.*, 2018 | [29386909](https://pubmed.ncbi.nlm.nih.gov/29386909/) |
|  |  |  | qacA_R | AATCCCACCTACTAAAGCAG |  | Kong *et al.*, 2018 | [29386909](https://pubmed.ncbi.nlm.nih.gov/29386909/) |
|  | *dfrK* | Dihydrofolate reductase | dfrK-F2 | CAAGAGATAAGGG**G**TTCAGC | 229 | Argudín *et al.*, 2015* | [25637268](https://pubmed.ncbi.nlm.nih.gov/25637268/) |
|  |  |  | dfrK-R2 | ACAGATACTTCGTTCCACTC |  | Argudín *et al.*, 2015* | [25637268](https://pubmed.ncbi.nlm.nih.gov/25637268/) |
|  | Staphylococcus species |  | STA_1 | CAGCTCGTGTCGTGAGATGT | 420 |  |  |
|  |  |  | STA_2 | AATCATTTGTCCCACCTCCG |  |  |  |
| Multiplex DQL | *dfrG* | Dihydrofolate reductase | dfrG-F | TTTCTTTGATTGCTGCGATG | 422 | Bertsch *et al.*, 2013 | [23344576](https://pubmed.ncbi.nlm.nih.gov/23344576/) |
|  |  |  | dfrG-R | CCCTTTTTGGGCAAATACCT |  | Bertsch *et al.*, 2013 | [23344576](https://pubmed.ncbi.nlm.nih.gov/23344576/) |
|  | *lsa(E)* | Lsa(E) ATP-binding cassette protein | lsaE_F | TTGTACGGAATGTATGG | 675 | Li *et al.,* 2013 | [23386262](https://pubmed.ncbi.nlm.nih.gov/23386262/) |
|  |  |  | lsaE_R | TTCGCTTCTATTAAGCACTCTT |  | Li *et al.,* 2013 | [23386262](https://pubmed.ncbi.nlm.nih.gov/23386262/) |
|  | *qacD* | efflux pump | qacC/D_F | GCCATAAGTACTGAAGTTATTGGA | 195 | Abd El-Aziz *et al.*, 2021 | [34206268](https://pubmed.ncbi.nlm.nih.gov/34206268/) |
|  |  |  | qacC/D_R | GACTACGGTTGTTAAGACTAAACCT |  | Abd El-Aziz *et al.*, 2021 | [34206268](https://pubmed.ncbi.nlm.nih.gov/34206268/) |
| Multiplex lincosamide | *lnu(A)* | Lincosamide nucleotidyltransferase | lnuA-F | GGTGGCTGGGGGGTAGATGTATTAACTGG | 323 | Moon *et al.*, 2015 | [25786036](https://pubmed.ncbi.nlm.nih.gov/25786036/) |
|  |  |  | lnuA-R | GCTTCTTTTGAAATACATGGTATTTTTCGATC |  | Moon *et al.*, 2015 | [25786036](https://pubmed.ncbi.nlm.nih.gov/25786036/) |
|  | *lnu(B)* | Lincosamide nucleotidyltransferase | lnuB-FOR | CCTAC**A**CTATTGTTTGTGGA**G** | 945 | Moon *et al.*, 2015* | [25786036](https://pubmed.ncbi.nlm.nih.gov/25786036/) |
|  |  |  | lnuB-REV | ATAACGTTACTCTCCTATTC |  | Moon *et al.*, 2015 | [25786036](https://pubmed.ncbi.nlm.nih.gov/25786036/) |
|  | *vga(A)V* | ATP-binding cassette protein | vga(A)V_F | CTCTTTGTACGAGTATATGG | 198 | Haroche *et al.*, 2000 | [10952567](https://pubmed.ncbi.nlm.nih.gov/10952567/) |
|  |  |  | vga(A)V_R | GTTTCTTAGTAGCTCGTTGAGC |  | Haroche *et al.*, 2000 | [10952567](https://pubmed.ncbi.nlm.nih.gov/10952567/) |
| Multiplex macrolide | *erm(A)* | rRNA methyltransferase | ermA1 | AAGCGGTAAACCCCTCTGA | 190 | Strommenger *et al.*, 2003 | [12958230](https://pubmed.ncbi.nlm.nih.gov/12958230/) |
|  |  |  | ermA2 | TTCGCAAATCCCTTCTCAAC |  | Strommenger *et al.*, 2003 | [12958230](https://pubmed.ncbi.nlm.nih.gov/12958230/) |
|  | *erm(C)* | rRNA methyltransferase | ermC1 | AATCGTCAATTCCTGCATGT | 299 | Strommenger *et al.*, 2003 | [12958230](https://pubmed.ncbi.nlm.nih.gov/12958230/) |
|  |  |  | ermC2 | TAATCGTGGAATACGGGTTTG |  | Strommenger *et al.*, 2003 | [12958230](https://pubmed.ncbi.nlm.nih.gov/12958230/) |
|  | *erm(B)* | rRNA methyltransferase | ermB_F | ACGACGAAACTGGCTAA | 409 | Mbindyo *et al.*, 2021 | [34202836](https://pubmed.ncbi.nlm.nih.gov/34202836/) |
|  |  |  | ermB_R | TGGTATGGCGGGTAA |  | Mbindyo *et al.*, 2021 | [34202836](https://pubmed.ncbi.nlm.nih.gov/34202836/) |
|  | *erm(T)* | rRNA methyltransferase | ermT_FW | GCTTGATAAAATTGGTTTTTGGA | 536 | Gómez-Sanz *et al.*, 2013 | [23629701](https://pubmed.ncbi.nlm.nih.gov/23629701/) |
|  |  |  | ermT_REV | ATTGGTTCAGGGAAAGGTC |  | Gómez-Sanz *et al.*, 2013 | [23629701](https://pubmed.ncbi.nlm.nih.gov/23629701/) |
|  | *msr(A)*** | efflux pump | msrA_F | GGCACAATAAGAGTGTTTAAAGG | 940 | Lina *et al*, 1999 | [10223914](https://pubmed.ncbi.nlm.nih.gov/10223914/) |
|  |  |  | msrA_R | AAGTTATATCATGAATAGATTGTCCTGTT |  | Lina *et al*, 1999 | [10223914](https://pubmed.ncbi.nlm.nih.gov/10223914/) |
| Multiplex phenicol | *fexA* | efflux pump | fexA-fw | GTACTTGTAGGTGCAATTACGGCTGA | 1272 | Kehrenberg & Schwarz, 2006 | [16569824](https://pubmed.ncbi.nlm.nih.gov/16569824/) |
|  |  |  | fexA-rv | CGCATCTGAGTAGGACATAGCGTC |  | Kehrenberg & Schwarz, 2006 | [16569824](https://pubmed.ncbi.nlm.nih.gov/16569824/) |
|  | *cat* | Chloramphénicol acetyltransférase | catpC221-F | ATTTATGCAATTATGGAAGTTG | 434 | Silva *et al.*, 2021 | [34359166](https://pubmed.ncbi.nlm.nih.gov/34359166/) |
|  |  |  | catpC221-R | TGAAGCATGGTAACCATCAC |  | Silva *et al.*, 2021 | [34359166](https://pubmed.ncbi.nlm.nih.gov/34359166/) |
|  | *cfr*** | rRNA methyltransferase | cfr-fw | TGAAGTATAAAGCAGGTTGGGAGTCA | 746 | Kehrenberg & Schwarz, 2006 | [16569824](https://pubmed.ncbi.nlm.nih.gov/16569824/) |
|  |  |  | cfr-rv | ACCATATAATTGACCACAAGCAGC |  | Kehrenberg & Schwarz, 2006 | [16569824](https://pubmed.ncbi.nlm.nih.gov/16569824/) |
| Multiplex tetracycline | *tet(K)* | efflux pump | tetK1 | GTAGCGACAATAGGTAATAGT | 360 | Strommenger *et al.*, 2003 | [12958230](https://pubmed.ncbi.nlm.nih.gov/12958230/) |
|  |  |  | tetK2 | GTAGTGACAATAAACCTCCTA |  | Strommenger *et al.*, 2003 | [12958230](https://pubmed.ncbi.nlm.nih.gov/12958230/) |
|  | *tet(M)* | ribosomal protection protein | tetM1 | AGTGGAGCGATTACAGAA | 158 | Strommenger *et al.*, 2003 | [12958230](https://pubmed.ncbi.nlm.nih.gov/12958230/) |
|  |  |  | tetM2 | CATATGTCCTGGCGTGTCTA |  | Strommenger *et al.*, 2003 | [12958230](https://pubmed.ncbi.nlm.nih.gov/12958230/) |
|  | *tet(L)* | efflux pump | tetL-rev | AACCAGCCAACTAATGACAATGAT | 1077 | Trzcinski *et al.*, 2000 | [10837427](https://pubmed.ncbi.nlm.nih.gov/10837427/) |
|  |  |  | tetL-up | ATAAATTGTTTCGGGTCGGTAAT |  | Trzcinski *et al.*, 2000 | [10837427](https://pubmed.ncbi.nlm.nih.gov/10837427/) |
|  | *tet(T)*** | ribosomal protection protein | tetT_fw | CAGTG**G**GAATATAAGGACACGTC | 644 | Katsarou *et al.*, 2021* | [33668332](https://pubmed.ncbi.nlm.nih.gov/33668332/) |
|  |  |  | tetT_rv | CAAGCCTTCTCTACAGCATC |  | Katsarou *et al.*, 2021 | [33668332](https://pubmed.ncbi.nlm.nih.gov/33668332/) |
|  | *mecA* | Penicillin-Binding Protein 2a | mecA-1 | GGGATCATAGCGTCATTATTC | 527 | Vannuffel *et al.*, 1998 | [9666026](https://pubmed.ncbi.nlm.nih.gov/9666026/) |
|  |  |  | mecA-2 | AACGATTGTGACACGATAGCC |  | Vannuffel *et al.*, 1998 | [9666026](https://pubmed.ncbi.nlm.nih.gov/9666026/) |

*The asterisk indicates that the oligonucleotide was modified compared to the one described in the article. The modified nucleotides are highlighted in bold.*

*The double asterisk indicates that the gene was not detected by PCR in the 329 tested isolates.*

**Table S3**

| ***rep* gene probe** | **Oligonucleotide name** | **Oligonucleotide sequence (5'-3')** | **Amplicon size (bp)** | **Reference** |
| --- | --- | --- | --- | --- |
| *rep5a* and *rep5c* | rep5F | CTTAAATCTACMTA**Y**TCWAAAMAYATGTT | 257 | [Lozano *et al.*, 2012](https://pubmed.ncbi.nlm.nih.gov/22685157/)* |
|  | rep5R | TCARCGTCAAAWGTRAACTCT |  |  |
| *rep7a* | rep7F | AGACGTAAYATGCGTRTTGA | 227 | [Lozano *et al.*, 2012](https://pubmed.ncbi.nlm.nih.gov/22685157/) |
|  | rep7R | CCAAAATAYTTDGTTTCTGG |  |  |
| *rep10* | rep10F | TATAAAGGCTCTCAGAGGCT | 383 | [Lozano *et al.*, 2012](https://pubmed.ncbi.nlm.nih.gov/22685157/) |
|  | rep10R | CCAAATTCGAGTAAGAGGTA |  |  |
| *rep13* | rep13F | ATGATGCAATATATTAAGCA | 403 | [Lozano *et al.*, 2012](https://pubmed.ncbi.nlm.nih.gov/22685157/) |
|  | rep13R | TACCAGAATAYTTAGCCATTTC |  |  |
| *rep15* | rep15F | CAGTAGAAGAAAATTATAAAGAAC | 327 | [Lozano *et al.*, 2012](https://pubmed.ncbi.nlm.nih.gov/22685157/) |
|  | rep15R | GTTATGGCTGGTTTTAATAAA |  |  |
| *rep16* | rep16F | CAGGAAAACACTTCGTTTAT | 592 | [Lozano *et al.*, 2012](https://pubmed.ncbi.nlm.nih.gov/22685157/) |
|  | rep16R | CTTCTATATCACTATCATTGTCATT |  |  |
| *rep19* | rep19F | GATGTTGAACCTTACGGCGAG | 287 | This study |
|  | rep19R | AGTTGTTCTGGGAATTCTTGCA |  |  |
| *rep20* | rep20F | CTAAATATTTRTTYGAAGATGC | 329 or 398 | [Lozano *et al.*, 2012](https://pubmed.ncbi.nlm.nih.gov/22685157/) |
|  | rep20R | CTGTGAATYTGGAAGTTCC |  |  |
| *rep21* | rep21F | TAGTTATCAAGCTCARARAG | 414 | [Lozano *et al.*, 2012](https://pubmed.ncbi.nlm.nih.gov/22685157/)* |
|  | rep21R | GMTTSWATRTCTTTATC**R**CC |  |  |
| *repUS5* | repUS5F | TCTTATATGGTGCGCTGGAGTA | 170 | This study |
|  | repUS5R | GCGTTATTTCTACCAACGCCA |  |  |
| *repUS12* | repUS12F | TTGTCCAATGTGCAACTGGAG | 193 | This study |
|  | repUS12R | TTGCATCATTCGGCGAAATCC |  |  |
| *repUS18* | repUS18F | G**R**ACGCTTCCTCAGCCGACT | 390 | [Lozano *et al.*, 2012](https://pubmed.ncbi.nlm.nih.gov/22685157/)* |
|  | repUS18R | ACCACTC**W**GGAAACTTCTCTTCTGC |  |  |
| *repUS20* | repUS20F | ACTTTACCAGCGAAAGCCAAA | 125 | This study |
|  | repUS20R | GAGAACAAACGCCCCTCATTA |  |  |
| *repUS76* | repUS76F | ATTGCYTACTGTATRTTTAGAGAYCG | 128 | This study |
|  | repUS76R | TGAGTTCCRCAATTTAAAAGWTYACAT |  |  |

*The asterisk indicates that the oligonucleotide was modified compared to the one described in the article. The modified nucleotides are highlighted in bold.*

**
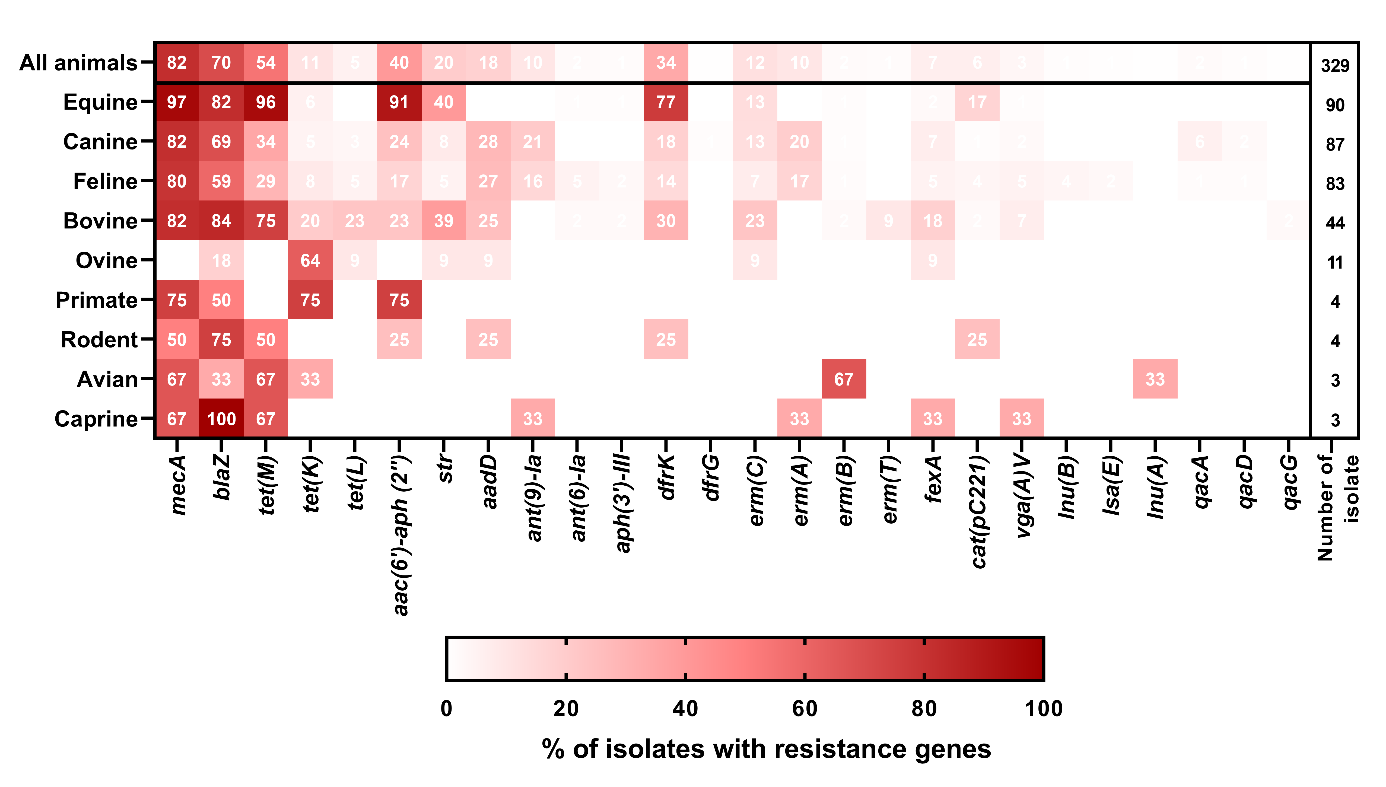
Figure S2**

**
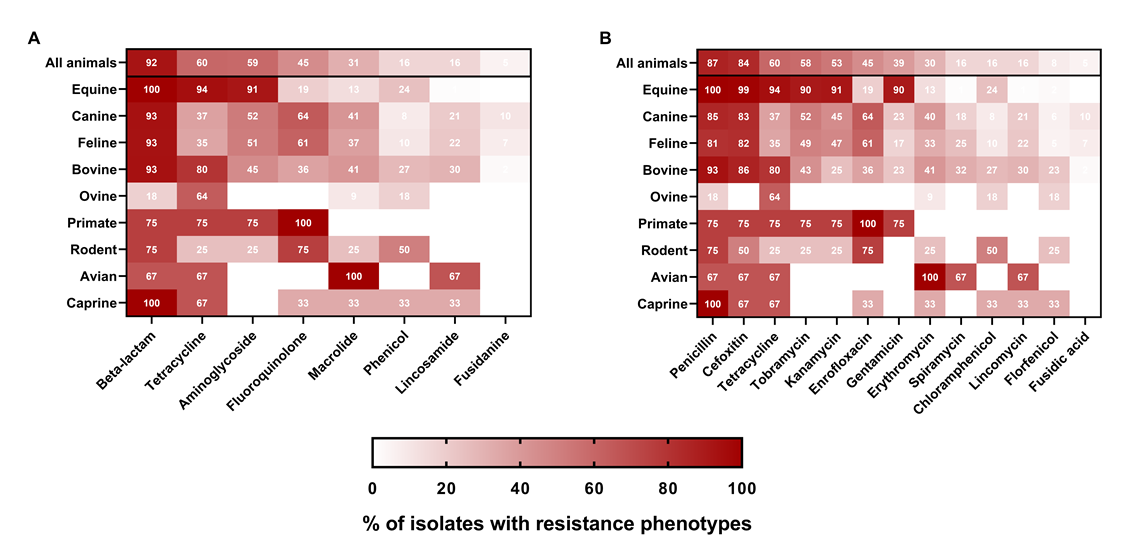
Figure S3**

**
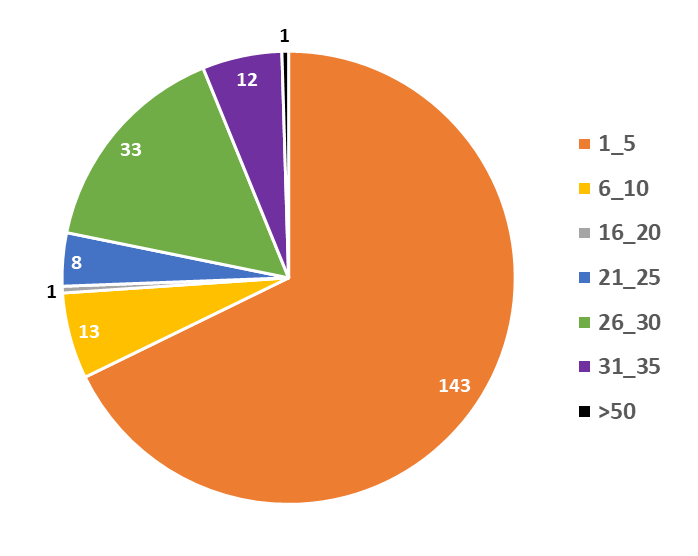
Figure S4**

**
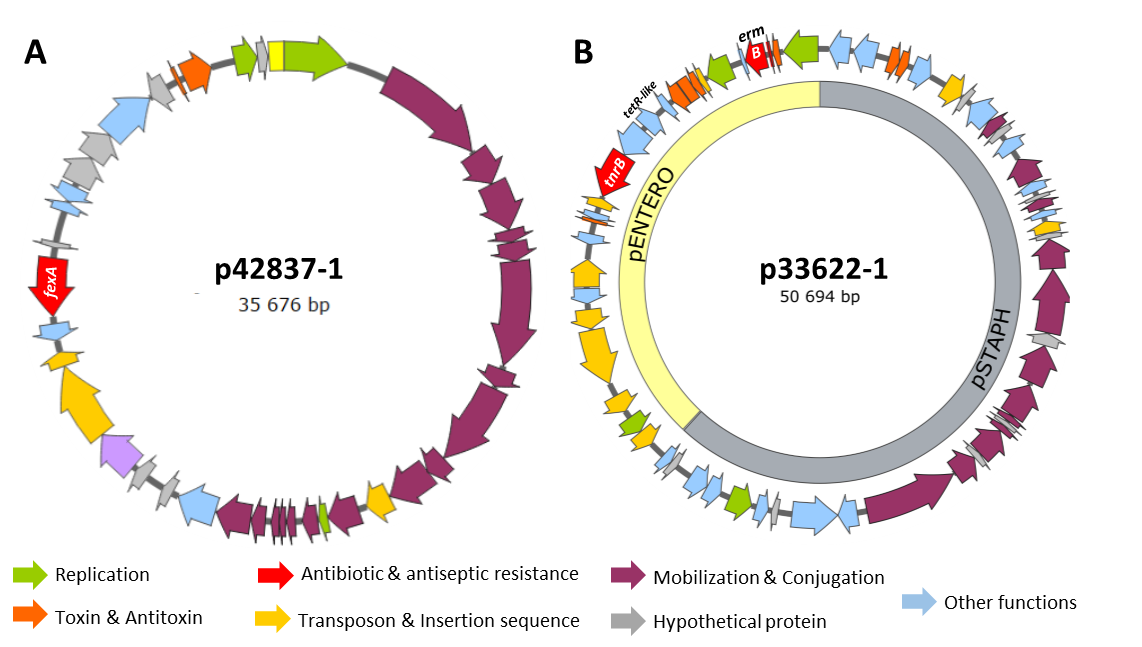
Figure S5**

**
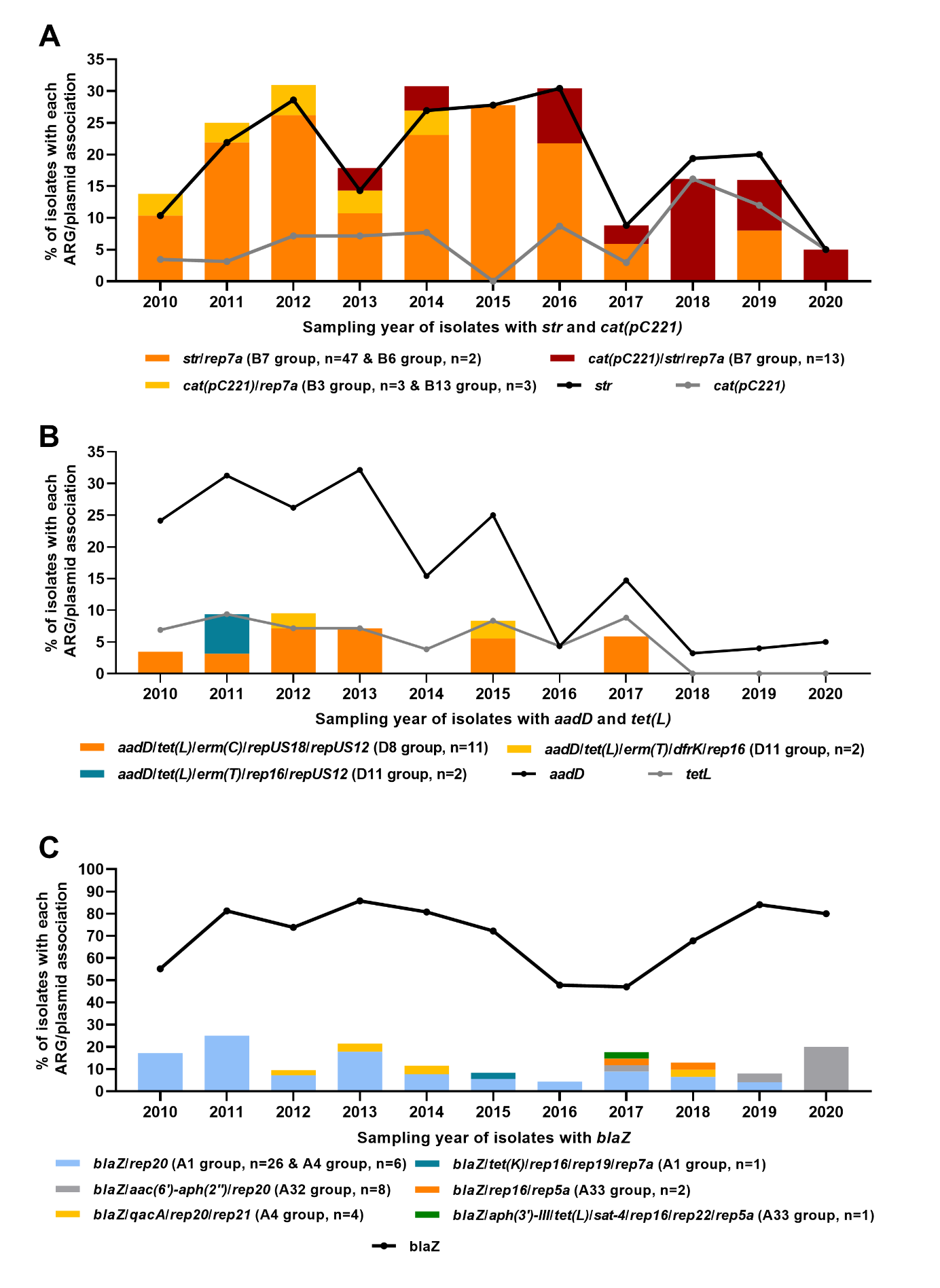
Figure S6**
